# Controlled ATRP Synthesis of Novel Linear-Dendritic Block Copolymers and Their Directed Self-Assembly in Breath Figure Arrays

**DOI:** 10.3390/polym11030539

**Published:** 2019-03-21

**Authors:** Xin Liu, Tina Monzavi, Ivan Gitsov

**Affiliations:** 1 Department of Chemistry, State University of New York–College of Environmental Science and Forestry, Syracuse, NY 13210, USA; xliu70@syr.edu; 2 Department of Chemistry and Biochemistry, University of South Carolina, Columbia, SC 29208, USA; tmonzavi@email.sc.edu; 3 The Michael M. Szwarc Polymer Research Institute, Syracuse, NY 13210, USA

**Keywords:** linear-dendritic copolymer, amphiphilic, self-assembly, ATRP, breath figure

## Abstract

Herein, we report the formation and characterization of novel amphiphilic linear-dendritic block copolymers (LDBCs) composed of hydrophilic dendritic poly(ether-ester), PEE, blocks and hydrophobic linear poly(styrene), PSt. The LDBCs are synthesized via controlled atom transfer radical polymerization (ATRP) initiated by a PEE macroinitiator. The copolymers formed have narrow molecular mass distributions and are designated as LGn-PSt M_n_, in which LG represents the PEE fragment, n denotes the generation of the dendron (*n* = 1–3), and M_n_ refers to the average molecular mass of the LDBC (*M*_n_ = 3.5–68 kDa). The obtained LDBCs are utilized to fabricate honeycomb films by a static “breath figure” (BF) technique. The copolymer composition strongly affects the film morphology. LDBCs bearing acetonide dendron end groups produce honeycomb films when the PEE fraction is lower than 20%. Pore uniformity increases as the PEE content decreases. For LDBCs with hydroxyl end groups, only the first generation LDBCs yield BF films, but with a significantly smaller pore size (0.23 μm vs. 1–2 μm, respectively). Although higher generation LDBCs with free hydroxyl end groups fail to generate honeycomb films by themselves, the use of a cosolvent or addition of homo PSt leads to BF films with a controllable pore size (3.7–0.42 μm), depending on the LDBC content. Palladium complexes within the two triazole groups in each of the dendron’s branching moieties can also fine-tune the morphology of the BF films.

## 1. Introduction

Polymer films with well-ordered micro- or nanosized pores are attractive to researchers due to their potential applications including, but not limited to, filters [1,2,3], sensors [4,5,6], cell culture media [7], photoelectronic devices [8,9], and templating materials [10]. In addition to the common “top down” approaches such as lithography [11], a directed self-assembly method, named the “breath figure” (BF) technique, draws intense attention due to its low cost and fast production rate. Even though the complete mechanism of BF formation is not fully elucidated, a widely accepted process for producing a BF array follows a common pattern. A polymer in a highly volatile solvent is cast on a substrate in a closed chamber with a high humidity. The surface of the polymer solution cools down as the solvent evaporates and promotes the condensation of water onto it. The water droplets grow in size, arrange into hexagonally packed arrays stabilized by a polymer precipitate, and eventually sink deeper into the polymer solution, subsequently serving as templates for ordered pores [12,13]. Since the introduction of the BF technique in 1994 by Francois [14], polymers with different architectures, including linear, star, comb-shaped, and dendritic, have been successfully employed to generate BF films [15]. Among them, amphiphilic block copolymers with poly(styrene), PSt, blocks have been studied intensively [15]. The hydrophilic block associates with water droplets to prevent their coalescence, while the PSt block forms the film framework with the potential for UV-radiation crosslinking to enhance its thermal and chemical resistance [16]. A vast collection of hydrophilic blocks, such as poly(acrylic acid) [17], poly(vinyl pyridine) [18], poly(ethylene oxide) [19], poly(N,N-dimethylaminoethyl methacrylate) [20], and dextran [21], have been used as the water-soluble component in copolymers. Copolymers with more compact dendritic hydrophilic blocks would provide an intriguing platform for BF film fabrication. For example, Connal et al. have achieved dramatic morphology tuning of BF films using core-crosslinked poly(styrene) stars with poly(ester) dendrons at the arm ends by manipulating the generation of dendrons and their peripheral groups [22]. Subsequently Hult’s group has demonstrated that block copolymers with linear-dendritic [23] or linear-hyperbranched architecture [24] can also generate porous films through a BF approach [25,26]. These BF films have been post-functionalized with fluorescent dyes or poly(ethylene glycol) and were evaluated for potential applications in patterned cell growth [27]. Our research group has recently synthesized a new family of hydrophilic poly(ether-ester), PEE, dendrons, based on 2,2-bis(methylol) propionic acid (bis-MPA) and oligo(ethylene glycol)s, which upon coupling with hydrophobic dendrons, could undergo a controlled self-assembly in aqueous media [28]. The present study is inspired by those previously published articles [22,23,24,25,26,27] and our own results [28], and targets the synthesis of LDBCs, composed of PEE dendrons and linear PSt blocks, which would be able to self-organize into BF films.

## 2. Materials and Methods

### 2.1. Materials

Azide-terminated poly(ether-ester), PEE, dendrons bearing triethylene glycol moieties (LGn-N_3_), were synthesized according to published procedures [28]. Styrene monomer was freshly distilled under vacuum before polymerization. Sodium Ascorbate, NaAsc, copper(I) bromide, CuBr; copper(II) sulfate, CuSO_4_; *N*,*N*,*N*′,*N*″,*N*″-pentamethyldiethylenetriamine, PMDTA, propargyl 2-bromoisobutyrate; and 2,5-dihydroxybenzoic acid (DHB) were all purchased from Sigma-Aldrich (St, Louis, MO, USA) and used as received. Trifluoroacetic acid was purchased from Alfa Aesar (Ward Hill, MA, USA) and used without further purification. Tetrahydrofuran (THF) was purchased from Fischer Scientific (Waltham, MA, USA) and distilled over NaOH. Dichloromethane (DCM) was purchased from Avantor (Center Vallley, PA, USA ) and used as received. Hexanes, methanol, and ethyl acetate were bought from Pharmoco-Aaper (Brookfield, CT, USA) and used as received.

### 2.2. Instrumentation

^1^H- and ^13^C NMR spectrums were recorded on the Bruker AVANCE 600 MHz NMR instrument (Bruker Corporation, Billerica, MA, USA) using an external lock and the trace of hydrogenated solvent signal as the internal reference.

The MALDI-TOF mass spectra were recorded on the Bruker Autoflex III MALDI-TOF instrument (Bruker Corporation, Billerica, MA, USA) using positive reflective mode with DHB as the matrix. The samples were prepared for analysis as follows: THF solutions of dendrons or copolymers (typically 1 mg/mL) were mixed with THF solutions of DHB (50 mg/mL), deposited on a stainless steel plate (MTP 384, Bruker), and air dried. 

Size exclusion chromatography (SEC) analyses were performed on a line consisting of a Waters M510 pump, a Waters U6K universal injector (Waters Corporation, Milford, MA, USA), three 5 μm PL Gel columns (50 Å, 500 Å, and Mixed C), and a Viscotek 250 dual refractive index/viscometry detector (Malvern Panalytical Inc., Westborough, MA, USA) The chromatograms were recorded at 40 °C and THF was used as the eluent at a 0.8 mL/min flow rate. A molecular mass calibration curve was generated using monodisperse poly(styrene) standards (162 Da−200 kDa, Polymer Standards Service, Warwick, RI, USA) and the relative molecular masses were calculated using Viscotek OmniSEC 3.1 software (Malvern Panalytical Inc., Westborough, MA, USA).

The interfacial tension between LDBCs in chloroform solution and water was measured by Ramé-hart goniometer Model 500 (Ramé-hart instrument co., Succasunna, NJ, USA) via pendant drop method and calculated by DROPimage advanced software (Ramé-hart instrument co.). The contact angle of water droplets on the polymer films was measured by the same instrument.

The cloud point was estimated by a turbidity test quantified by the transmission of the polymer solution at 500 nm using the Beckman DU640B spectrometer (Beckman Coulter Inc., Jersey City, NJ, USA). The cloud point was established when the transmission was 50% lower than the original solution as water was added.

The surface morphology of the BF films was characterized by scanning electron microscopy (SEM) performed on the JEOL JSM100 instrument (JEOL USA, Peabody, MA, USA) with a 10–20 kV accelerating voltage and 8–10 mm working distance. The samples were coated with Au/Pd by Denton Desk V sputter coater before observation under the microscope.

### 2.3. Synthesis of Dendritic Macroinitiators by “Click” Chemistry

The LGn-N_3_ (n denotes the generation number) and propargyl 2-bromoisobutyrate were dissolved in 1 mL of THF. To the above solution, 10 mg of CuSO_4_ in 150 μL of H_2_O and 20 mg of NaAsc in 150 μL of water were added. The reaction mixture was stirred vigorously at room temperature for 5 min. The completion of the reaction was confirmed by MALDI-TOF showing the absence of unreacted LGn-N_3_. The reaction mixture was diluted with 5 mL of water and then extracted three times with 5 mL of ethyl acetate. The combined organic layers were dried and concentrated under reduced pressure. The crude product was purified by silica gel chromatography using ethyl acetate and methanol as solvents (10:1 gradually changing to 4:1). The dendritic macroinitiators were isolated as colorless viscous oils. They were stored at −4 °C for further usage as DCM solutions (typically 20 mg/mL). 

#### 2.3.1. First Generation PEE Dendron Initiator LG1-i

LG1-N_3_ (300 mg, 0.29 mmol), propargyl 2-bromoisobutyrate (300 mg, 1.46 mmol), 10 mg CuSO_4_ and 20 mg NaAsc were used. Yield: 279 mg, 77.2%.

^1^H NMR (600 MHz, CDCl_3_) δ: 7.84 (s, 1H), 7.69 (s, 2H), 5.34 (s, 2H), 4.62 (s, 4H), 4.57–4.53 (m, 6H), 4.31 (t, 4H, J = 5Hz), 4.24 (t, 2H, J = 4.9Hz), 4.20 (d, 4H, J = 11.6Hz), 3.90-3.87 (m, 6H), 3.69 (t, 4H, J = 4.9Hz), 3.67–3.62 (m, 18H), 3.59 (s, 4H), 1.94 (s, 6H), 1.44 (s, 6H), 1.39 (s, 6H), 1.21 (s, 6H), 1.19 (s, 3H) ppm, Figure S1A.

^13^C HMR (150 MHz, CDCl_3_) δ: 174.13, 171.41, 144.97, 124.85, 123.55, 98.07, 72.15, 70.60, 70.58, 70.47, 69.51, 69.40, 69.09, 69.05, 65.97, 64.99, 63.77, 63.63, 59.24, 55.70, 50.31, 50.16, 48.32, 41.85, 30.69, 24.58, 22.73, 18.67, 17.91 ppm, Figure S1B.

MALDI-TOF calculated for C_52_H_84_BrN_9_O_20_ [M] *m*/*z* = 1233.50; found [M+H]^+^ *m*/*z* = 1234.446 [M+Na]^+^ *m*/*z* = 1256.735, Figure S2.

SEC: *M*_n_ = 900, Ð = 1.01

#### 2.3.2. Second Generation PEE Dendron Initiator LG2-i

LG2-N_3_ (300 mg, 0.124 mmol), propargyl 2-bromoisobutyrate (300 mg, 1.46 mmol), 10 mg CuSO_4_ and 20 mg NaAsc were used. Yield: 261 mg, 80.3%.

^1^H NMR (600 MHz, CDCl_3_) δ: 7.84 (s, 1H), 7.71–7.69 (m, 6H), 5.33 (s, 2H), 4.61 (s, 8H), 4.61 (s, 4H), 4.56–4.52 (m, 14H), 4.31 (t, 8H, J = 5 Hz), 4.24–4.22 (m, 6H), 4.20 (d, 8H, J = 11.6 Hz), 3.90–3.87 (m, 14H), 3.69 (t, 8H, J = 4.9 Hz), 3.67–3.59 (m, 56H), 1.94 (s, 6H), 1.44 (s, 12H), 1.39 (s, 12H), 1.21 (s, 12H), 1.19 (s, 9H) ppm, Figure S3A.

^13^C NMR (150 MHz, CDCl_3_) δ:174.21, 174.12, 144.94, 124.86, 123.56, 98.05, 72.19, 72.12, 70.56, 70.52, 70.47, 70.44, 69.50, 69.38, 69.05, 65.96, 64.97, 63.77, 63.61, 59.24, 53.41, 50.31, 50.16, 48.33, 48.29, 41.85, 30.69, 24.60, 22.73, 18.67, 17.92 ppm, Figure S3B.

MALDI-TOF calculated for C_114_H_184_BrN_21_O_44_ [M] *m*/*z* = 2630.73; found [M+H]^+^ *m*/*z* = 2631.971; [M+Na]^+^ *m*/*z* = 2653.982, Figure S4.

SEC: *M*_n_ = 2010, Ð = 1.01.

#### 2.3.3. Third Generation PEE Dendron Initiator LG3-i

LG3-N_3_ (350 mg, 0.067 mmol), propargyl 2-bromoisobutyrate (300 mg, 1.46 mmol), 10 mg CuSO_4_ and 20 mg NaAsc were used. Yield: 303 mg, 84.1%. 

^1^H NMR (600 MHz, CDCl_3_) δ: 7.69 (s, 15H), 5.33 (s, 2H), 4.61 (s, 16H), 4.60 (s, 12H), 4.55–4.52 (m, 30H), 4.30 (t, 16H, J = 4.9 Hz), 4.24-4.21 (m, 14H), 4.19 (d, 16H, J = 11.6 Hz), 3.89-3.87 (m, 30H), 3.69 (t, 16H, J = 4.9 Hz), 3.66-3.58 (m, 122H), 1.39 (s, 6H), 1.44 (s, 24H), 1.38 (s, 24H), 1.21 (s, 24H), 1.18 (s, 12H), 1.18(s, 9H) ppm, Figure S5A. 

^13^C HMR (150MHz, CDCl_3_) δ: 174.22, 174.13, 144.94, 144.89, 142.05, 124.88, 123.58, 98.06, 72.24, 72.10, 70.56, 70.52, 70.47, 70.44, 69.50, 69.38, 69.04, 65.96, 64.97, 63.78, 63.61, 59.24, 55.75, 53.41, 50.31, 50.15, 50.11, 48.35, 48.29, 41.85, 30.69, 24.63, 22.70, 18.66, 17.92 ppm, Figure S5B. 

MALDI-TOF calculated for C_238_H_384_BrN_45_O_92_ [M] *m*/*z* = 5427.60; found [M+H]^+^ *m*/*z* = 5428.594, Figure S6.

SEC: *M*_n_ = 4500, Ð = 1.01.

### 2.4. Synthesis of Linear-Dendritic block Copolymers by Dendron Initiated ATRP

A desired amount of CuBr was charged into a Schlenk flask with a stir bar and then vacuumed and refilled three times with argon. LGn-i and PMTDA were dissolved in the styrene monomer. The mixture was degassed by three freeze-thaw cycles. Upon warming to room temperature, the mixture was transferred into the flask charged with CuBr via a syringe. The mixture was stirred at room temperature for 15 min to form the copper(I)-PMDTA complex. Then, the Schlenk flask was immersed into an oil bath preheated to 95 °C. The polymerization was stopped at different times, and the mixture was diluted with 15 mL of THF and passed through a basic alumina column. Another 15 mL of THF was used to rinse the column. The polymers were isolated by precipitation of the combined THF fraction into hexanes. The precipitation process was repeated three times to completely remove the unreacted monomer and initiator.

### 2.5. Breath Figure Film Preparation

The breath figure films were produced by a static method adopted from the literature [17]. Briefly, a piece of glass was mounted on the top of a steel stub by double sided tape and placed into a 50 mL jar. The jar was filled with 5 mL deionized water, sealed with a rubber stopper, and placed into a 25 °C water bath. After 15 min, water droplets condensed on the wall of the jar, indicating that the interior of the jar was saturated with water vapors (~100% relative humidity). Following this, 20 μL of polymer solution in CHCl_3_ (20 mg/mL) was cast onto the glass slide inside the jar using a syringe inserted into the rubber stopper. After 15 min, the glass with the BF film on it was removed from the jar and air dried overnight.

## 3. Results and Discussion

**Scheme 1:**
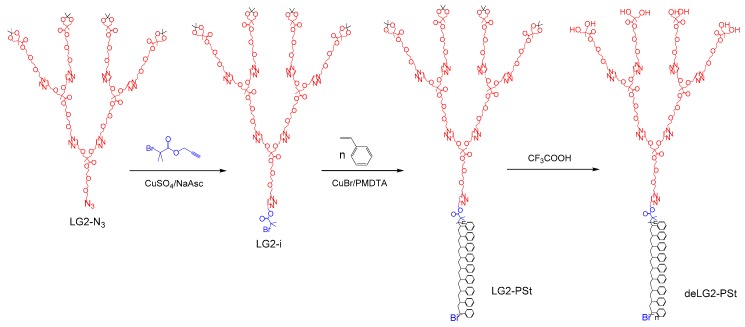
Synthesis of LDBCs by LG2 Dendron-Initiated Atom Transfer Polymerization.

There are three strategies to synthesize LBDCs, including: (a) coupling previously prepared dendron(s) to complementary functionalized linear chain end(s); (b) stepwise dendron growth from a linear polymer end group; and (c) dendron initiated controlled polymerization [29]. The initial attempt to prepare PEE-*block*-PSt LDBCs was conducted by the coupling strategy (a) using PEE dendrons with an azide at the focal point and alkyne terminated poly(styrene), Scheme S1. However, the LDBCs obtained by this method possessed a bimodal molecular mass distribution, as revealed by size-exclusion chromatography (SEC), Figure S7. The broadened dispersity (Ð) was caused by “oxidative” coupling, as reported in our previous study [28] and also observed by other groups [30]. To obtain LDBCs with a narrow molecular mass distribution, the “dendron first” (c) strategy was adopted. An example is shown in Scheme 1.

**Figure 1:**
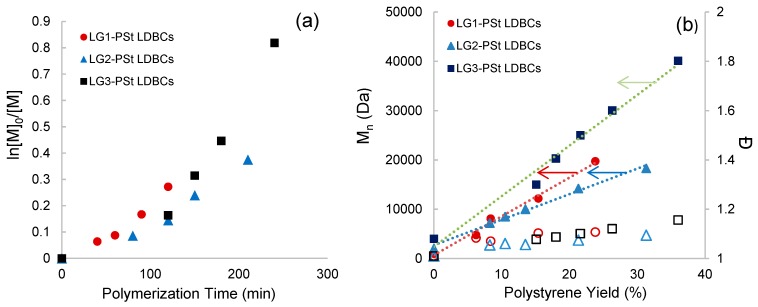
(**a**) Kinetic plots of dendron-initiated ATRP; (**b**) dependence of molecular mass, M_n_ (solid symbols), and polydispersity, PDI (hollow symbols), on PSt yield. ATRP conditions: all polymerizations were performed in bulk at 95 °C; [LG1-i]:[St]:[CuBr]:[PMDTEA] = 1:300:2:2; [LG2-i]:[St]:[CuBr]:[PMDTEA] = 1:400:2:2; [LG3-i]:[St]:[CuBr]:[PMDTEA] = 1:1000:4:4.

**Figure 2:**
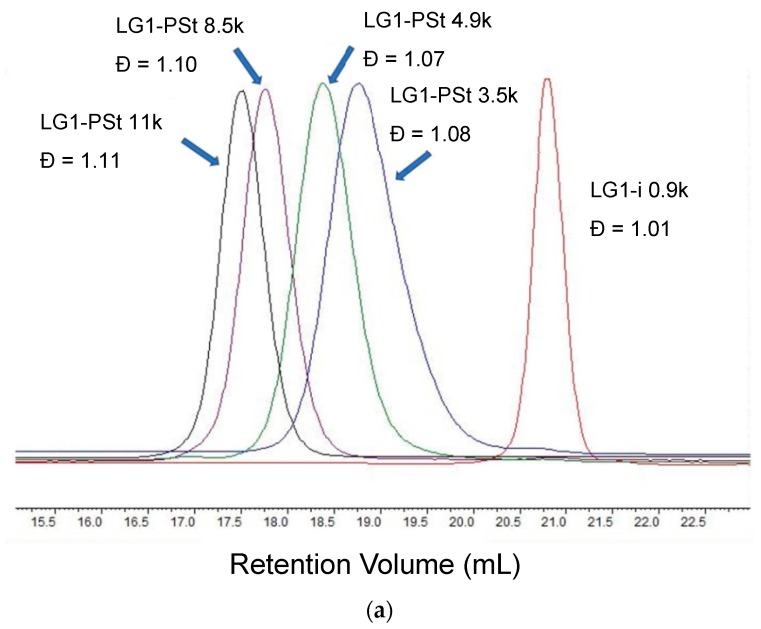

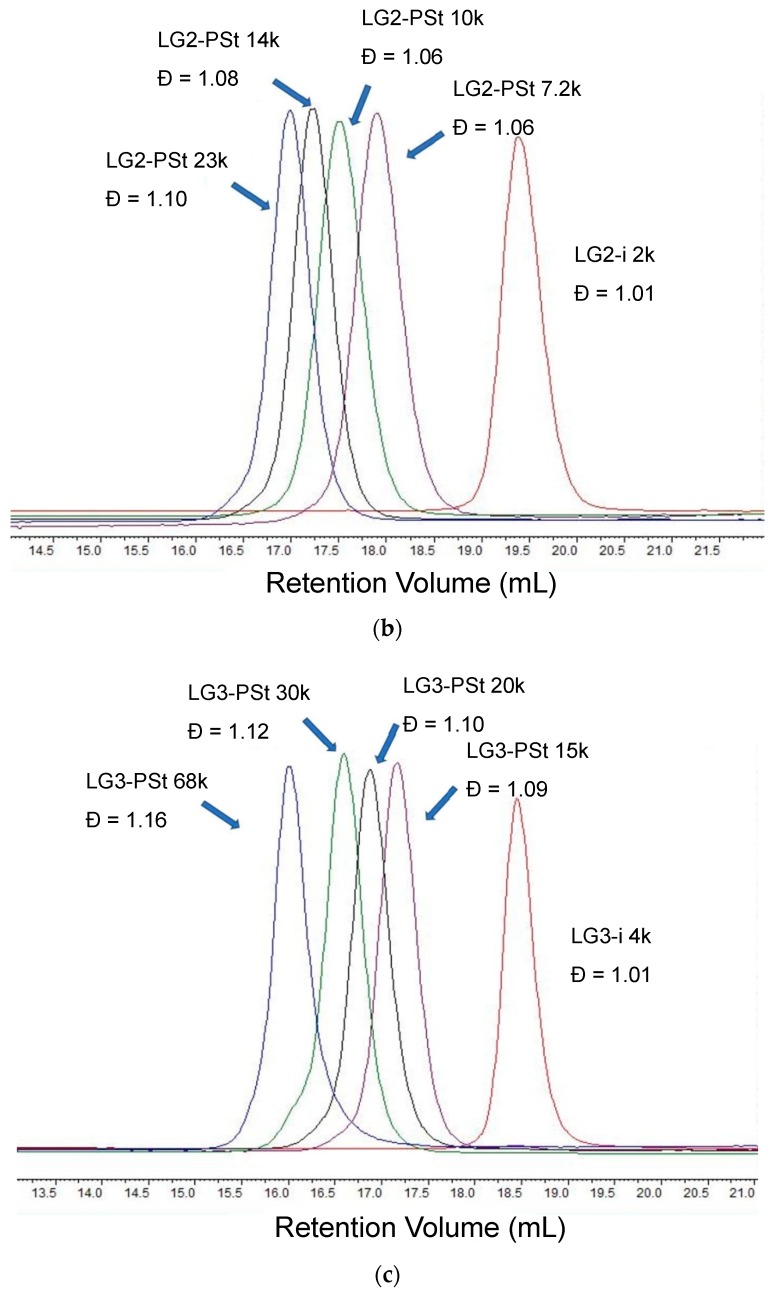
(**a**) SEC traces of LG1-PSt copolymers with different lengths of the PSt block, SEC traces of LG2-PSt (**b**), and LG3-PSt (**c**) LDBCs with different lengths of the PSt block.

The dendron initiators were prepared by “click” chemistry coupling of propargyl 2-*iso*-bromobutyrate to an azide-terminated dendron. The poly(styrene) block was then grown by atom transfer radical polymerization (ATRP) at 95 °C, a common temperature for styrene ATRP [31]. Since both the macroinitiator and the target polymer are soluble in styrene, the polymerization was performed in bulk. The molecular mass of the PSt block was controlled by changing the ATRP reaction time and catalyst concentration. The kinetic study of these polymerizations is shown in Figure 1. The conversion rate decreased with the increase of the macroinitiator generation (size). In fact, the polymerization initiated by LG3-i was very slow if the same molar ratio of catalyst to initiator as in LG1-i and LG2-i was used—only 15% conversion was achieved after 4 h of polymerization. That is why the molar ratio of catalyst to initiator was doubled for LG3-i to boost the polymerization rate (these results are shown in Figure 1a). All three processes proceeded in a typical controlled radical polymerization fashion with slow initiation and no termination and chain transfer [32] - the molecular mass increased linearly with conversion and Ð remained relatively low (<1.15), Figure 1b and Figure 2. The initiator efficiency (*f*) decreased with the increase in dendron generation, probably due to the steric factors hindering a monomer approach to the reactive site [33] (calculation of *f* is described in SI and Figure S9). The estimated values decreased in the following order: *f_LG1_*(81–93%), *f_LG2_*(66–79%), *f_LG3_*(46–67%). The loss of initiator efficiency was evidenced by SEC with traces of unreacted dendrons clearly visible (an example with LG2-i initiated polymerization is shown in Figure S8). Remarkably, this initiator portion did not initiate polymerization at later stages, otherwise the Ð of the final product would have been much higher. The unreacted initiator fraction along with the copper catalyst was removed by activated alumina.

The SEC curves of the isolated LDBCs with acetonide protective end groups are shown in Figure 2. The polymers were treated with trifluoroacetic acid to produce the LDBCs with hydroxyl end groups. The completion of the acetonide removal was confirmed by NMR (Figure S10). It should be noted that the procedure did not affect the molecular mass distribution of the resulting copolymer, which remained very narrow, Figure S11.

**Figure 3:**
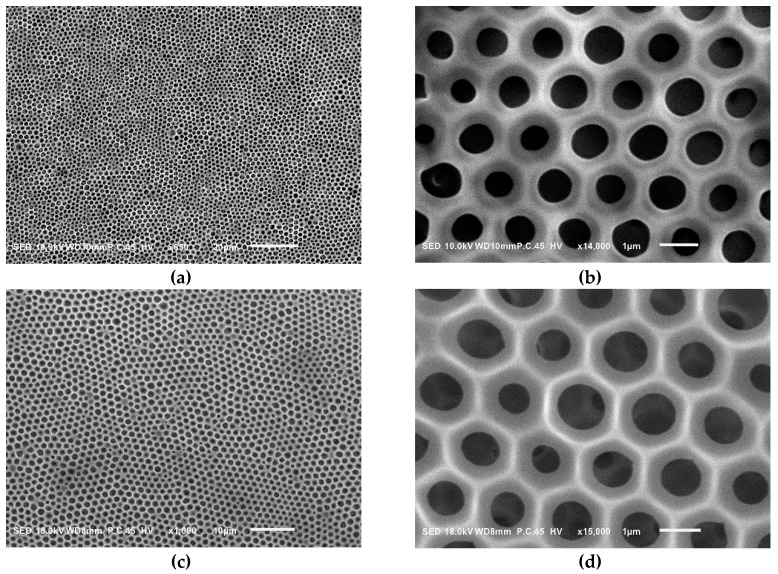

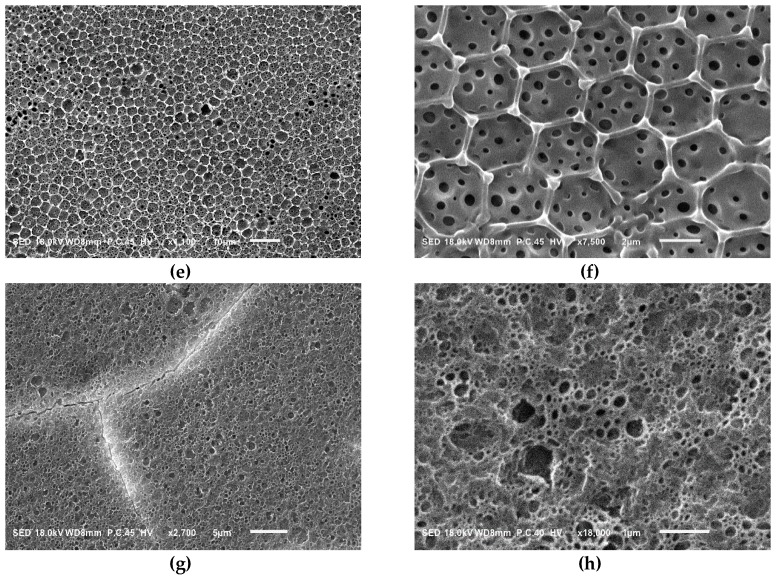
SEM images of BF films prepared from first generation LDBCs with acetonide end groups: (**a**,**b**) LG1-PSt 11k; (**c**,**d**) LG1-PSt 8.5k**.** SEM images of BF films prepared from first generation LDBCs with acetonide end groups: (**e**,**f**) LG1-PSt 4.9k, edge of the film; (**g**,**h**) LG1-PSt 3.5k. Scale bars: (**a**) 20 μm; (**c**,**e**) 10 μm; (**g**) 5 μm, (**f**) 2 μm; (**b**,**d**,**h**) 1 μm.

The obtained LDBCs were employed to produce porous films by the BF technique. The morphology of BF films is affected by polymer composition and casting conditions, such as air flow rate and relative humidity. The variability in the casting parameters could be minimized by choosing a static BF method instead of the conventional dynamic approach. Since the purpose of this report was mainly focused on the effects of polymer composition on the film morphology, the static BF method was adopted, in which the CHCl_3_ polymer solution was cast on a glass substrate at 25 °C in a closed chamber saturated with water vapors. It was suggested that the hydrophilic/hydrophobic balance in amphiphilic block copolymers is a key parameter in BF film production [15,20]. Thus, the effect of F_PEE_ was firstly investigated in LG1-PSt series (F_PEE_ refers to the weight fraction of the hydrophilic PEE dendron). The acetonide-protected PEE LG1 dendron is not water-soluble, but the interfacial tension between the chloroform polymer solution and water is lower than the tension between pure CHCl_3_ and water (measured as 30.5 mN·m^−1^) and further decreases with higher PEE generations. This trend could be explained with the increased content of hydrophilic triethylene glycol (triEG) moieties in the dendrons at the interface between CHCl_3_ and the condensing water. It is known that the reduced interfacial tension facilitates the stabilization of condensed water droplets through the association of the hydrophilic parts of the polymers [15,20]. The morphologies of obtained BF films were investigated by scanning electron microscopy (SEM), Figure 3. Not surprisingly, LG1-PSt LDBCs with relatively low F_PEE_ (LG1-PSt 8.5k and LG1-PSt 11k) and relatively long PSt tails (compared to LG1) were able to generate honeycomb films with fairly good regularity. Two specific features in this morphology were observed: hexagonally packed frameworks and cyclic open pores, Figure 3b,d (light domains and dark areas, respectively), very similar to BF films from core-crosslinked star poly(styrene) with polyester dendron end groups [22]. The average pore diameters were 0.85 μm and 0.9 μm for LG1-PSt 11k and 8.5k, respectively. As the hydrophilicity of LDBC increased (shorter PSt chain), a unique morphology was discovered in BF films of LG1-PSt 4.9k - a random porous layer extended beneath hexagonally packed arrays with some defects, Figure 3e,f. The diameter of the hexagonal skeleton was ~3μm, while the pore sizes in the underlying layer were ranging from 0.2 to 0.6 μm. This interesting morphology was only detected at the films’ periphery, while the other areas of the films exhibited very irregular porous patterns (see also LG2-PSt 10k in Figure S13g,h). Water droplets of different sizes condensing at the surface, sinking, and migrating during multiple successive stages from the center of the solution toward the periphery most probably led to this hierarchical pattern [34], which was also reported without a discussion of other polymer systems, including branched polymers with a relatively high hydrophilicity [22]. As expected, a further decrease in PSt chain length led to a completely irregular porous morphology, as observed in BF LG1-PSt 3.5k films, Figure 3g,h and Figure S12.

The observed behavior hints at another important factor in BF film formation: the precipitation rate of the copolymers. The cloud point experiments showed that the precipitation rate of the LDBCs decreased with shorter PSt chains. Slow polymer precipitation would allow droplets coalescence. The interesting morphology observed at the edges of BF surfaces formed with LG1-PSt 4.9k, Figure 3e,f, was probably aided by this phenomenon. On the contrary, fast polymer precipitation around the condensing water droplets would most probably prevent their coalescence, leading to more regular patterns.

**Figure 4:**
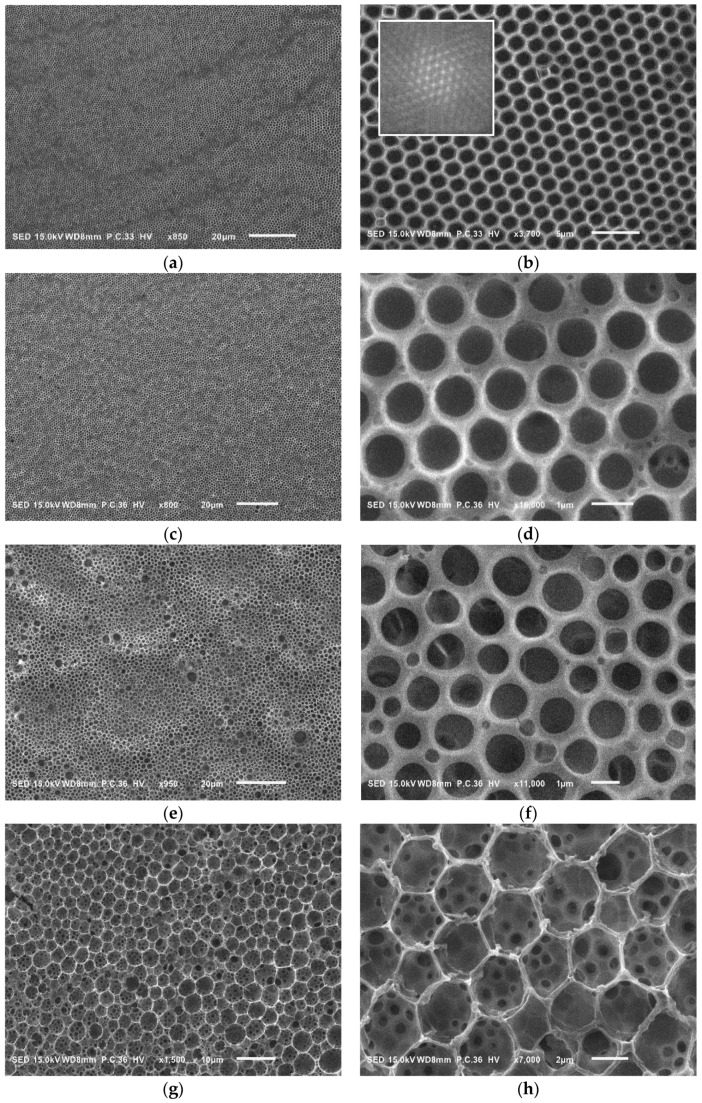
SEM images of BF films prepared from third-generation LDBCs with acetonide end groups: (**a**,**b**) LG3-PSt 68k, FFT - insert in image (**b**); (**c**,**d**) LG3-PSt 30k; (**e**,**f**) LG3-PSt 20k - majority portion of the film; (**g**,**h**) edge of LG3-PSt 20k film. Scale bars: (**a**,**c**,**e**) 20 μm; (**g**) 10 μm; (**b**) 5 μm; (**h**) 2 μm; (**d**,**f**) 1 μm.

Taking into account the results from LG1-PSt series, the second and third generation of LDBCs with various F_PEE_ were then chosen to prepare BF films. Figure 4 contains images from the SEM characterization of LG3-PSt BF films. A similar morphology transition trend was also exhibited by LG2-PSt LDBCs, Figure S13. In accordance with the previous results, LG2-PSt and LG3-PSt with low F_PEE_ (<15%) were able to produce honeycomb films with regular patterns. Especially LG3-PSt 68k, LDBCs with the longest poly(styrene) tail, formed uniform honeycombs of 1.1 μm pores with long range order, Figure 4a,b. The Fast Fourier transformation (FFT) of the image confirmed the regular arrangement of the main framework (insert in Figure 4b). The observed regularity could be attributed to the moderately reduced interfacial tension and the fast precipitation rate. With an F_PEE_ slightly above 20%, LG3-PSt 20k still produced BF films, although with a greatly reduced uniformity of pores. Again, the slower precipitation rate of the shorter PSt block induced the formation of hexagonal packed frameworks with underlying porous films, observed on the edge of the LG3-PSt 20k BF films (Figure 4g,h), similar to the previously mentioned patterns from LG1-PSt 4.9k (Figure 3e,f).

**Figure 5:**
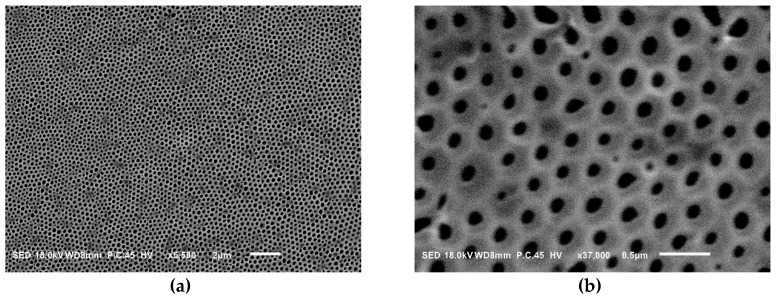

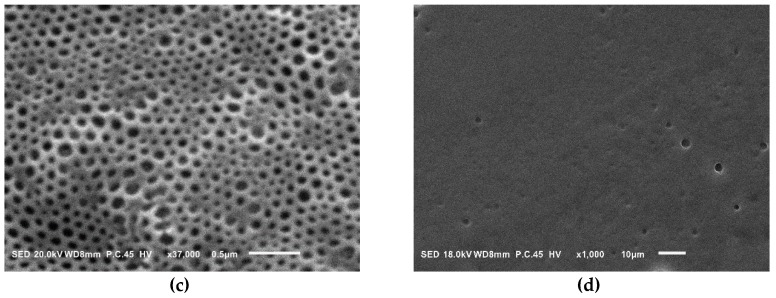
SEM images of BF films produced from first generation LDBCs with hydroxyl end groups: (**a**, **b**) deLG1-PSt 11k; (**c**) deLG1-PSt 8.5k; (**d**) deLG1-PSt 4.9k. Scale bars: (**a**) 2 μm; (**b**, **c**) 0.5 μm; (**d**) 10 μm.

The LDBCs with hydroxyl groups at the dendron periphery have notably higher surface activity manifested by the lower surface tension of their solutions (Table 1, columns 3 and 4). They were also investigated in BF film fabrications starting with LG1-PSt series. The SEM micrographs of the obtained BF films surface morphologies are shown in Figure 5. In distinction to its acetonide-protected counterpart, deLG1-PSt 11k produced much smaller pores with a ~0.16 μm diameter. Unlike LG1-PSt, deLG1-PSt has a smaller dendron footprint due to the removal of the bulky protecting groups and the potential hydrogen bonding between the free peripheral hydroxyl groups in the PEE dendron. Moreover, these hydroxyl groups have an increasing tendency to form hydrogen bonds with the condensing water molecules, thus reducing the interfacial tension between the polymer CHCl_3_ solution and water droplets. These two factors are probably instrumental for the surfactant-like polymer to be able to stabilize the small water droplets and result in a larger interfacial area, leading to the formation of smaller pores [35]. It was noticed that some pores were not spherical, Figure 5b. Such non-spherical pores usually result from water droplets coalescence due to the reduced precipitation rate of a more hydrophilic polymer. With increased hydrophilic content, deLG1-PSt 8.5k generated porous films with an even smaller pore size (~0.08 μm), clearly resulting from the previously discussed lower surface tension. As hydrophilicity increased even further, deLG1-PSt 4.9k formed films with smooth, almost pore-free, surfaces, Figure 5d.

Similar to deLG1-PSt 4.9k, the second- and third-generation LDBCs with hydroxyl end groups all failed to generate a porous morphology by the static BF technique, regardless of the length of the PSt tail. This phenomenon can be explained by the spreading coefficient (*S*), defined as the ability of a water droplet to spread on the polymer solution [36]:

(1) $$S=\gamma_{s}-\left(\gamma_{w}+\gamma_{sw}\right)$$

where $\gamma_{s}$ is the surface tension of polymer solution, $\gamma_{w}$ is the surface tension of condensed water droplets, and $\gamma_{sw}$ is the interfacial tension between the polymer solution and water. If *S* < 0, the water droplets will form a lens on the surface of the polymer solution. If *S* > 0, the water droplets will spread. The $\gamma_{sw}$ parameter has a negative effect on *S*: low interfacial tension will cause the water droplets to spread and thus can no longer serve as templates for porous films [20].

It should be mentioned that the deprotected second and third PEE dendrons were no longer soluble in CHCl_3_, and therefore would selectively align and aggregate on the solution surface, rendering it hydrophilic (low $\gamma_{sw})$. When the water droplets condense on this hydrophilic surface, they spread over the entire area and coalesce. Notably, at low concentrations (1 mg/mL), the hydroxyl-terminated LDBCs formed reverse micelles in CHCl_3_ solutions, which display a strong Tyndall effect (Figure S14). These micelles were highly hydrophilic and might absorb large amounts of water and fuse instead of surrounding the water droplets [25].

**Figure 6:**
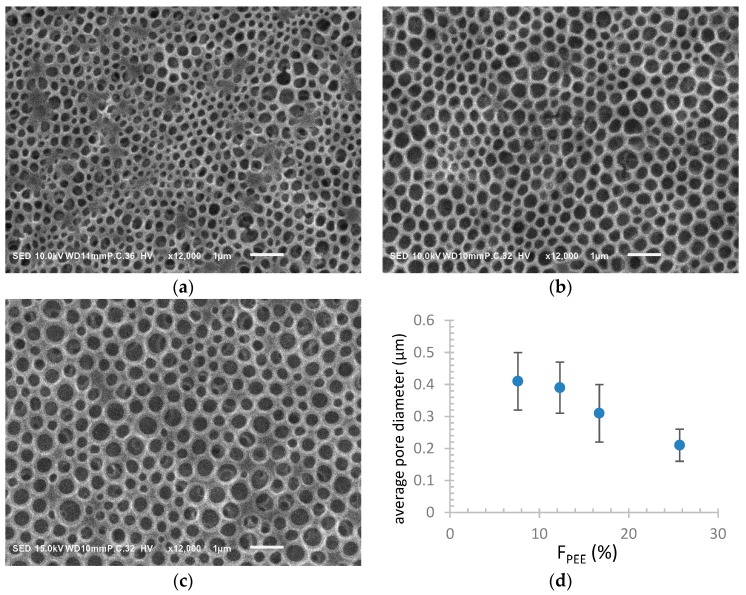
SEM images of BF films of third-generation LDBCs with hydroxyl end groups from THF/CHCl_3_ solutions (1:3 *v*/*v*): (**a**) deLG3-PSt 20k; (**b**) deLG3-PSt 30k; (**c**) deLG3-PSt 68k. (**d**) Plot of average pore size vs. F_PEE_.

The mechanism of BF film formation requires the use of water immiscible solvents to dissolve the polymer. Very interestingly, when THF (a water miscible solvent) was used as a cosolvent, BF films were successfully produced by the deprotected second- and third-generation LDBCs, which failed in the same process using chloroform as the only solvent. THF is a good solvent for the deprotected PEE block, which was demonstrated by turbidity tests. The deprotected second- and third-generation PEE dendrons formed a milky suspension in chloroform, which became clear upon adding THF. This means that adding THF to LDBC chloroform solution brought the insoluble PEE blocks from the surface back into the bulk solution. Presumably, the net result was an increase in the interfacial tension which prevented the water droplets from spreading. Unfortunately, the attempt to measure the interfacial tension between the THF/chloroform solution of LDBCs and water failed because an emulsion immediately formed when the polymer solution was placed in water (Figure S15). The emulsion was created by the THF-enabled phase transfer of LDBCs to stabilize chloroform droplets. The disruption of the surface alignment of the higher-generation dendrons of the LDBCs in chloroform by adding THF could be evidenced by the increase of the contact angle (CA) of water droplets on the polymer film air dried from pure chloroform and air dried from THF/CHCl_3_. The films formed from both solutions were smooth and therefore, the chemical composition of the surface would be the main contribution factor for the measured CA. For example, the CA on the LG3-PSt 68k film dried from chloroform was 14.5°, suggesting a high PEE content on the surface, while the CA increased to 75° if 25% THF was added, indicating a decrease of the PEE content on the surface (Figure S16). Furthermore, the inverse micelles were no longer present (no clear Tyndall effect, as shown in Figure S15). Thus, the problem of water take-up by the inverse micelles as discussed above was also circumvented. All of these factors seemingly contributed to the successful BF film assembly. SEM images of BF films prepared from the third-generation LBDCs are presented in Figure 6. Noticeably, the pores are not well-arranged due to the relatively large hydrophilic domains in the polymers and the water miscible cosolvent. For the same reason, the average pore diameter had a weak dependence on F_PEE_, as presented in Figure 6d.

**Figure 7:**
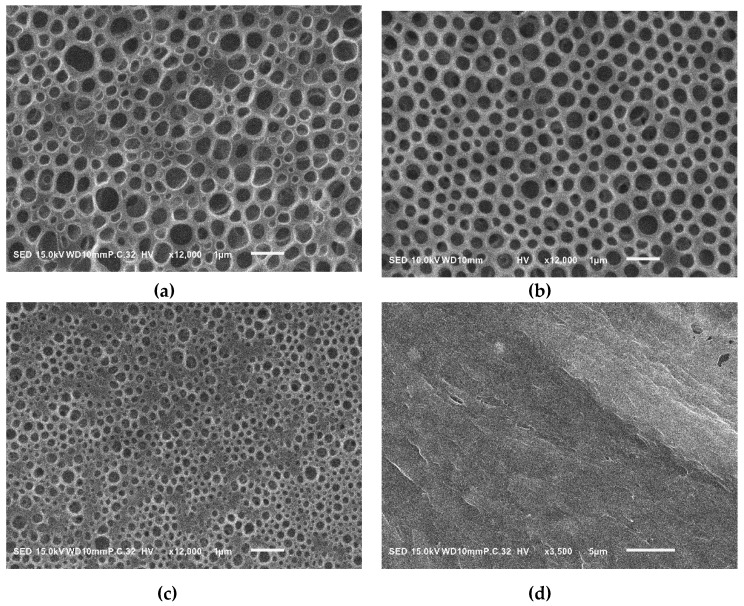
SEM images of BF films produced from deLG3-PSt 68k with different THF contents in the copolymer chloroform solution: (**a**) 10%; (**b**) 25%; (**c**) 50%; (**d**) 75%.

It seems that the content of THF added in the chloroform polymer solution could possibly have an assessable effect on the surface morphology of BF films. LG3-PSt 68k was chosen to investigate this effect, with the THF content varying from 10% to 75%. The corresponding SEM images of the films are shown in Figure 7. When the content of THF was low, a very irregular porous pattern was generated from the uncontrolled coalescence of water droplets, Figure 7a. A BF film with some pore regularity was produced at 25% THF content, Figure 7b. As the THF amount increased further to 50%, a porous film was formed with a significantly reduced pore size, Figure 7c. Finally, when the content of THF reached 75%, the film had no pores, Figure 7d. The observed behavior could be explained by the good miscibility of THF with water, which causes efficient spreading of the condensing water over the entire surface, with no pores being formed.

**Figure 8:**
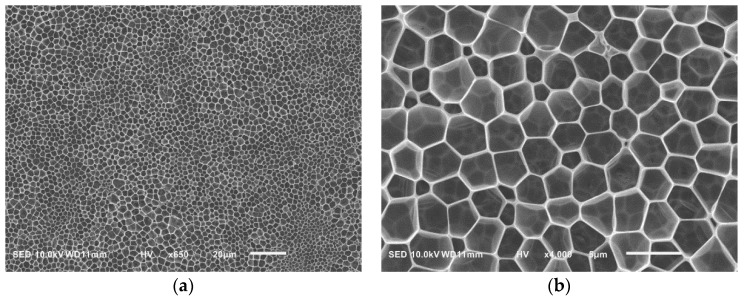
SEM images of BF film produced by deLG3-PSt 68k using 1:3 methanol/chloroform mixed solvent.

Acetone, acetonitrile, and methanol were also investigated as possible cosolvents to produce BF films. Acetone and acetonitrile generated similar film morphologies as THF (Figure S17), while methanol induced a completely new morphology consisting of irregular polygon arrays (1 to 3μm in diameter), shown in Figure 8a,b.

In this pattern resembling a Voronoi diagram [37], the number of neighboring polygonal pores to the center one ranges from four to eight, as estimated from the binary image process using ‘image J’ (Figure S18). The Voronoi number, defined as the average number of neighboring cells (i.e., pores), is six in this BF film. The origin of this unique pattern cannot be explained at this moment. It would be reasonable to assume that it might be caused by the much slower evaporation rate of methanol compared to chloroform, resulting in a longer evaporation time of the entire solution. In that way, the growth time of the water droplets would be extended, leading to micrometer scale pores compared to the sub-micrometer scale pores created by other cosolvents with a similar evaporation time to chloroform.

**Figure 9:**
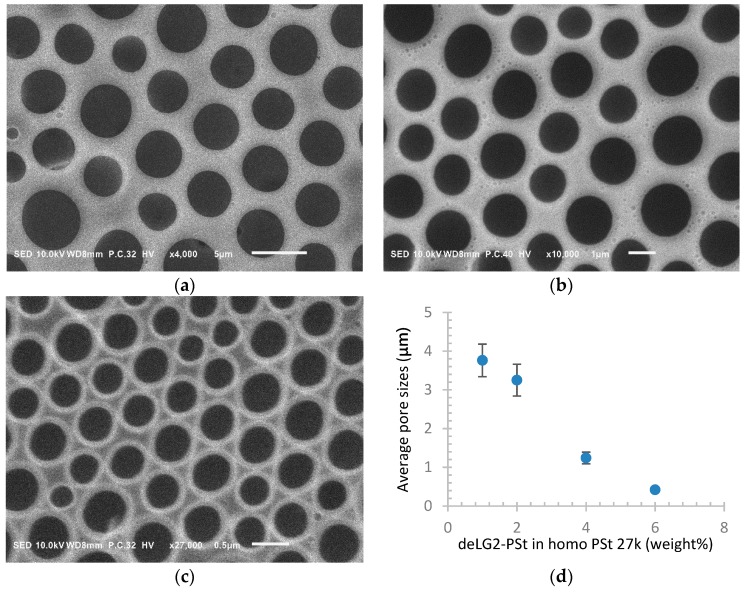
SEM images of BF films produced from deLG2-PSt 23k and homo PSt 27k at different LDBC concentrations: (**a**) 0.1 wt%, (**b**) 0.4 wt%, (**c**) 0.6 w t%. (**d**) Plot of average pore diameter vs. deLG2-PSt content.

Polymer blends are often used in the BF techniques to achieve a different morphology or functionality [38,39]. Another useful strategy to tune the BF film pore size employs a surfactant as an additive to homopolymers [40] or nanoparticles [41]. It is known that the hydrophilic component promotes the nucleation of water droplets while the water condensation rate remains constant. The overall outcome is that the pore sizes decrease with the surfactant concentration. The deprotected LDBCs could serve as polymeric surfactants to achieve pore size control in BF films. To verify this hypothesis, deLG2-PSt 23k was added to homo PSt 27k at concentrations ranging between 0.1 to 0.8 wt%. As shown in Figure 9, the average diameter of the pores decreased from ~3.7 μm to ~0.4 μm with the increase of LDBC concentration. Notably, these pores were not well-organized (see also Figure S19 for more micrographs of these BF films). When the deLG2-PSt content reached 0.8 wt%, a non-porous film was formed, possibly due to the high hydrophilic content on the solution surface, as previously discussed. Unlike the BF films with multiple layer pores produced by pure LDBCs, the polymer blends generated a single layer of pores. The SEM images of fractured BF films prepared by LG2-PSt, deLG2-PSt, and deLG2-PSt blended with homo PSt are provided in Figure S20. It was demonstrated that the low content of the hydrophilic domain in the polymer blend could lead to pores organized in a single layer, while multiple layers could be caused by a large hydrophilic fraction [42].

**Scheme 2:**
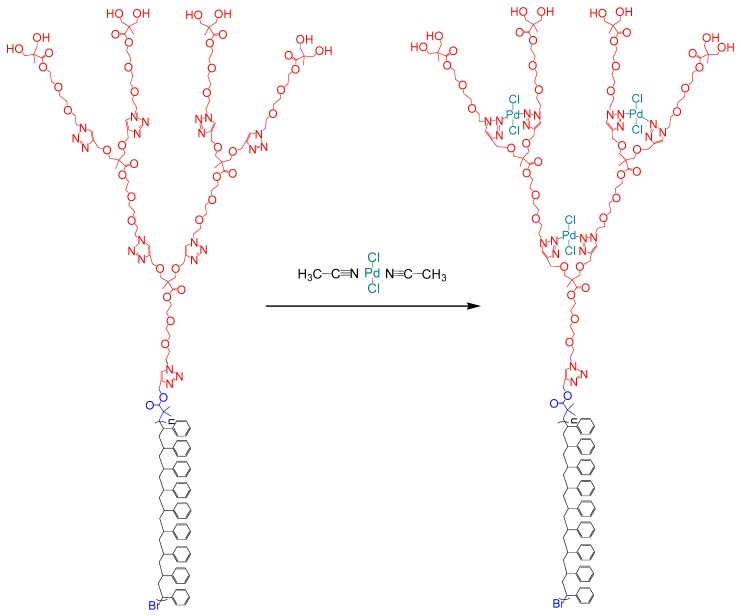
Formation of de-LG2 LDBC palladium complexes through ligand exchange.

Chemical modification on the triazole rings in the copolymers is an intriguing strategy to adjust their physical properties. Recently, Ji et al. reported that the ionization of the triazole rings in a rod-coil block copolymer can influence the BF film morphology [43]. As previously demonstrated, the triazole rings at the branching point of the PEE dendrons can form complexes with palladium [28]. An added aim of this study was to explore whether a palladium (Pd) complex would affect the morphology of BF films, Scheme 2. Firstly, the Pd complex of deLG1-PSt 11k was investigated in a BF process.

**Figure 10:**
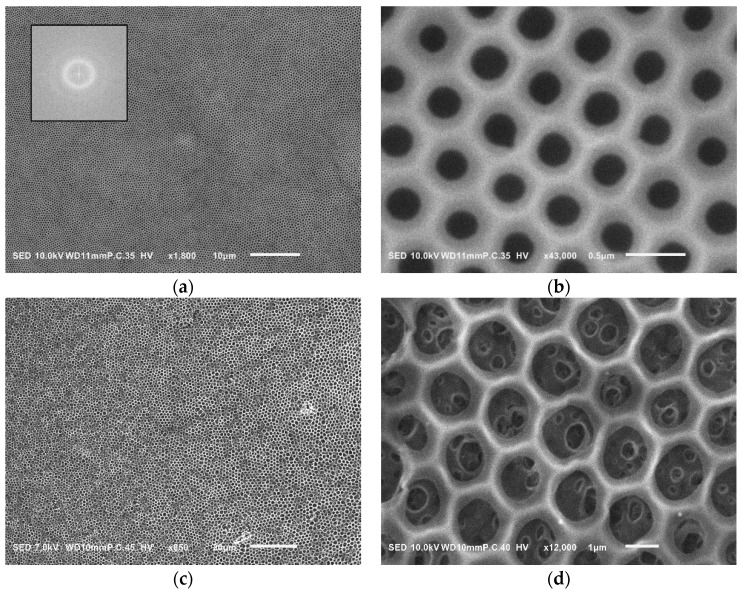
SEM images of BF films generated by Pd-LDBC complexes. (**a**,**b**) Pd-deLG1-PSt 11k, insert: FFT of the image; (**c**,**d**): Pd-deLG2-PSt 23k.

The regularity of the pores was greatly improved compared to the copolymer in non-complexed form (Figure 10a,b). The FFT of the image demonstrated pores in short range order. Noticeably, the average pore diameter increased from ~0.16 μm to ~0.29 μm due to the slightly diminished surfactant properties (the interfacial tension increases by 1.2 mN·m^−1^ after Pd complexing). Very surprisingly, the Pd complex of deLG2-PSt 23k (Scheme 2) produced BF films in pure chloroform without any cosolvent. The surface morphology of the obtained BF film is presented in Figure 10c,d. A slight increase of interfacial tension by 0.9 mN·m^−1^ was also observed. Apparently, the PEE-Pd complex was less water-soluble, preventing the inverse micelles from absorbing water in chloroform and thus stabilizing water droplets instead. The attempts to fabricate BF films with the Pd complex of deLG3-PSt LDBCs were unsuccessful due to the high-water solubility of the third-generation PEE-Pd complex.

## 4. Conclusions

In summary, a series of novel linear-dendritic block copolymers containing poly(ether-ester), PEE, dendrons of generations 1-3 and PSt linear chains of a molecular mass between 3.5 and 68 kDa were successfully synthesized by dendron-initiated ATRP. The efficiency of the macroinitiators was affected by the size of the dendron, and all copolymers formed had a monomodal and narrow molecular mass distribution. The obtained LDBCs were employed to produce porous films by the static breath figure technique. It was found that the fraction of PEE (F_PEE_) and the nature of the peripheral dendritic groups had a significant effect on the size of the pores (0.08–3.7 μm) and on the surface morphology of the BF films. The acetonide-functionalized LDBCs with low F_PEE_ (<20%) generated more uniform honeycomb structures in comparison to those with high F_PEE_. Once the acetonide groups were removed, only the first-generation LDBCs with low F_PEE_ (deLG1-PSt 11k and 8.5k) were able to produce BF films with much smaller pore sizes. Interestingly, BF films were formed with a cosolvent added to the chloroform solution. In addition, when deLG2-PSt 23k was mixed with homo poly(styrene) of a comparable molecular mass, BF films with controllable pore sizes were produced, depending on the LDBC content. Finally, palladium complexing between the triazole rings in the PEE dendrons provided an alternative way to adjust the morphology of the resulting BF films. Potential applications of those films include site-specific binding and catalysis.

## Figures and Tables

**Table 1:** Summary of BF film surface morphology produced by LDBCs.

Sample ^a^	F_PEE_^b^ (%)	γ ^c^ (mN m^−1^)	γ’ ^d^ (mN m^−1^)	Cloud Point ^e^ (%)	Pore Size (μm)	Pore Regularity
LG1-PSt 11k	9.2	29.7	23.3	18.7	0.85 ± 0.15	fair
LG1-PSt 8.5k	12.6	29.5	21.4	20.0	0.90 ± 0.12	fair
LG1-PSt 4.9k	21.5	29.1	18.1	28.5	0.40 ± 0.20	poor
LG1-PSt 3.5k	27.8	29.2	18.6	31.0	-	very poor
LG2-PSt 23k	10.2	28.6	16.1	18.0	1.10 ± 0.11	fair
LG2-PSt 14k	16.9	27.9	15.7	19.4	1.02 ± 0.06	fair
LG2-PSt 10k	23.0	28.5	13.2	21.5	0.84 ± 0.42	poor
LG3-PSt 68k	7.6	26.5	17.9	11.5	1.1	very good
LG3-PSt 30k	16.7	25.2	16.5	16.6	0.95 ± 0.06	good
LG3-PSt 20k	25.7	24.4	12.3	20.0	1.14 ± 0.30	poor

^a^ sample name: LGn-PSt M_n_, where (n) is the generation of PEE dendron and (M_n_) is the number average molecular mass of the LDBC in kDa, measured by SEC; ^b^ weigh fraction of PEE dendron in the LDBCs estimated by ^1^H NMR; ^c^ interfacial tension between water and acetonide-protected LDBCs in chloroform solution; ^d^ interfacial tension between water and deprotected LDBCs in chloroform solution; ^e^ water content in THF solution of acetonide-protected LDBC when the cloud point is reached.

## Supplementary Materials

The following are available online at https://www.mdpi.com/2073-4360/11/3/539/s1: ^1^H- and ^13^C NMR spectra, MALDI-TOF spectra, coupling synthesis of linear-dendritic copolymers, calculation of initiator efficiency, size-exclusion chromatography eluograms, and SEM images. Figure S1. ^1^H NMR (**A**) and ^13^C NMR (**B**) of the first-generation PEE macroinitiator in CDCl_3_. Figure S2. MALDI-TOF of the first-generation PEE macroinitiator. Figure S3. ^1^H NMR (**A**) and ^13^C NMR (**B**) of the second-generation PEE macroinitiator in CDCl_3_. Figure S4. MALDI-TOF of the second-generation PEE macroinitiator. Figure S5. ^1^H NMR (**A**) and ^13^C NMR (**B**) of the third-generation PEE macroinitiator in CDCl_3_. Figure S6. MALDI-TOF of the third-generation PEE macroinitiator. Scheme S1 Synthesis of LDBC by a “coupling” method. Figure S7. SEC trace of LDBC prepared by a “coupling” method. The obtained LDBC is a mixture of normal “click” and oxidative coupling product. Figure S8. SEC monitoring the LG2-i initiated polymerization progress with time. The small peak appearing in the lower molecular mass region is the unreacted dendron initiator. Figure S9. ^1^H NMR spectrum of LG2-PSt 10k as an example of how to calculate FPEE. Figure S10. NMR of LG3-PSt 20k before (**A**) and after (**B**) the acetonide protecting group is removed, as highlighted. The signals of CH_3_ groups on acetonide at 1.44 and 1.38 (marked as *) ppm are completely removed after the deprotection step. Figure S11. SEC traces of LG3-PSt 20k before (**A**) and after deprotection (**B**) (deLG3-PSt 20k). Figure S12. SEM image of BF film prepared by LG1-PSt 4.9k. Rough surface with 0.2–0.6 μm pores irregularly distributed. Figure S13. SEM images of BF films prepared by the second-generation LDBCs with acetonide end groups: (**a**, **b**) LG2-PSt 23k; (**c**, **d**) LG2-PSt 14k; (**e**, **f**) LG2-PSt 10k, major portion of the film; (**g**, **h**) LG2-PSt 10k, edge of the film. Figure S14. Tyndall effect of inverse micelles formed by LDBCs with hydroxyl end groups in chloroform (vial on the left side) and inverse micelles are dissolved by adding 25% THF (vial on the right side): (**a**) deLG2-PSt 23k; (**b**) deLG3-PSt 68k. Figure S15. Emulsion formed by placing LDBCs with hydroxyl end groups in THF/chloroform solution added to water: (**a**) deLG2-PSt 23k; (**b**) deLG3-PSt 68k. Figure S16. Contact angle measurement of water droplet on the polymer film prepared by air dry film drop casted on glass surface in different solvents: (**a**) pure chloroform; (**b**) 1:3 THF/chloroform. The LDBC used is deLG3-PSt 68k. Figure S17. SEM images of BF films prepared by deLG3-PSt 68k in mixed solvents: (**a**,**b**) 1:3 acetone/chloroform; (**c**,**d**) 1:3 acetonitrile/chloroform. Figure S18. Binary image obtained from Figure 8 using ImageJ to estimate the Voronoi number. Figure S19. SEM images of BF films prepared by homo-PSt mixed with deLG2-PSt 23k at different concentrations: (**a**) 0.1 wt%; (**b**) 0.2 wt%; (**c**) 0.4 w t%; (**d**) 0.6 wt%. Figure S20. SEM images of fractured BF films produced by: (**a**) LG2-PSt 23k; (**b**) deLG2-PSt 23k; (**c**) deLG2-PSt 23k and homo-PSt blends.

## References

[1] Täuber K., Zimathies A., Yuan J. (2015). Porous Membranes Built Up from Hydrophilic Poly(ionic liquid)s. Macromol. Rapid Commun..

[2] Yu B., Cong H., Li Z., Yuan H., Peng Q., Chi M., Yang S., Yang R., Wickramasinghe S.R., Tang J. (2015). Fabrication of highly ordered porous membranes of cellulose triacetate on ice substrates using breath figure method. J. Polym. Sci. Part B Polym. Phys..

[3] Du C., Zhang A., Bai H., Li L. (2013). Robust Microsieves with Excellent Solvent Resistance: Cross-Linkage of Perforated Polymer Films with Honeycomb Structure. Acs Macro Lett..

[4] Li T., Li L., Sun H., Xu Y., Wang X., Luo H., Liu Z., Zhang T. (2017). Porous Ionic Membrane Based Flexible Humidity Sensor and its Multifunctional Applications. Adv. Sci..

[5] Böker A., Lin Y., Chiapperini K., Horowitz R., Thompson M., Carreon V., Xu T., Abetz C., Skaff H., Dinsmore A.D. (2004). Hierarchical nanoparticle assemblies formed by decorating breath figures. Nat. Mater..

[6] Chen P.-C., Wan L.-S., Ke B.-B., Xu Z.-K. (2011). Honeycomb-Patterned Film Segregated with Phenylboronic Acid for Glucose Sensing. Langmuir.

[7] Yin H., Bulteau A., Feng Y., Billon L. (2016). CO_2_-Induced Tunable and Reversible Surface Wettability of Honeycomb Structured Porous Films for Cell Adhesion. Adv. Mater. Interfaces.

[8] Wang J., Wang C.-F., Shen H.-X., Chen S. (2010). Quantum-dot-embedded ionomer-derived films with ordered honeycomb structures via breath figures. Chem. Commun..

[9] Yin S., Zhang Y., Kong J., Zou C., Li C.M., Lu X., Ma J., Boey F.Y.C., Chen X. (2011). Assembly of Graphene Sheets into Hierarchical Structures for High-Performance Energy Storage. ACS Nano.

[10] De Boer B., Stalmach U., Nijland H., Hadziioannou G. (2000). Microporous Honeycomb-Structured Films of Semiconducting Block Copolymers and Their Use as Patterned Templates. Adv. Mater..

[11] Cao T., Wei F., Jiao X., Chen J., Liao W., Zhao X., Cao W. (2003). Micropatterns of Protein and Conducting Polymer Molecules Fabricated by Layer-by-Layer Self-Assembly and Photolithography Techniques. Langmuir.

[12] Hernández-Guerrero M., Stenzel M.H. (2012). Stenzel, Honeycomb structured polymer films via breath figures. Polym. Chem..

[13] Zhang A., Bai H., Li L. (2015). Breath Figure: A Nature-Inspired Preparation Method for Ordered Porous Films. Chem. Rev..

[14] Widawski G., Rawiso M., François B. (1994). Self-organized honeycomb morphology of star-polymer polystyrene films. Nature.

[15] Stenzel M.H., Barner-Kowollik C., Davis T.P., Barner-Kowollik C. (2006). Formation of honeycomb-structured, porous films via breath figures with different polymer architectures. J. Polym. Sci. A Polym. Chem..

[16] Li L., Zhong Y., Li J., Chen C., Zhang A., Xu J., Ma Z. (2009). Thermally stable and solvent resistant honeycomb structured polystyrene films via photochemical cross-linking. J. Mater. Chem..

[17] Li L., Chen C., Zhang A., Liu X., Cui K., Huang J., Ma Z., Han Z. (2009). Fabrication of robust honeycomb polymer films: A facile photochemical cross-linking process. J. Colloid Interface Sci..

[18] Escalé P., Rubatat L., Derail C., Save M., Billon L. (2011). pH Sensitive Hierarchically Self-Organized Bioinspired Films. Macromol. Rapid Commun..

[19] Park J.S., Lee S.H., Han T.H., Kim S.O., Lee J. (2007). Hierarchically Ordered Polymer Films by Templated Organization of Aqueous Droplets. Adv. Funct. Mater..

[20] Wu B.-H., Zhu L.-W., Ou Y., Tang W., Wan L.-S., Xu Z.-K. (2015). Systematic Investigation on the Formation of Honeycomb-Patterned Porous Films from Amphiphilic Block Copolymers. J. Phys. Chem. C.

[21] Chen S., Alves M.-H., Save M., Billon L. (2014). Synthesis of amphiphilic diblock copolymers derived from renewable dextran by nitroxide mediated polymerization: Towards hierarchically structured honeycomb porous films. Polym. Chem..

[22] Connal L.A., Vestberg R., Hawker C.J., Qiao G.G. (2008). Dramatic Morphology Control in the Fabrication of Porous Polymer Films. Adv. Funct. Mater..

[23] Gitsov I. (2008). Hybrid linear dendritic macromolecules: From synthesis to applications. J. Polym. Sci. A Polym. Chem..

[24] Wurm F., Frey H. (2011). Linear–dendritic block copolymers: The state of the art and exciting perspectives. Prog. Polym. Sci..

[25] Andrén O.C.J., Walter M.V., Yang T., Hult A., Malkoch M. (2013). Multifunctional Poly(ethylene glycol): Synthesis, Characterization, and Potential Applications of Dendritic–Linear–Dendritic Block Copolymer Hybrids. Macromolecules.

[26] Walter M.V., Lundberg P., Hult D., Hult A., Malkoch M. (2013). A one component methodology for the fabrication of honeycomb films from biocompatible amphiphilic block copolymer hybrids: A linear–dendritic–linear twist. Polym. Chem..

[27] Mongkhontreerat S., Walter M.V., Cai Y., Brismar H., Hult A., Malkoch M. (2015). Functional porous membranes from amorphous linear dendritic polyester hybrids. Polym. Chem..

[28] Liu X., Gitsov I. (2018). Thermosensitive Amphiphilic Janus Dendrimers with Embedded Metal Binding Sites. Synthesis and Self-Assembly. Macromolecules.

[29] Gitsov I. (2015). Synthesis and Self-Assembly of Linear-Dendritic Hybrid Polymers. Encyclopedia of Polymeric Nanomaterials.

[30] Angell Y., Burgess K. (2007). Base Dependence in Copper-Catalyzed Huisgen Reactions: Efficient Formation of Bistriazoles. Angew. Chem..

[31] Matyjaszewski K., Xia J. (2001). Atom Transfer Radical Polymerization. Chem. Rev..

[32] Elias H.G. (1997). An Introduction to Polymer Science.

[33] Connal L.A., Vestberg R., Hawker C.J., Qiao G.G. (2007). Synthesis of Dendron Functionalized Core Cross-linked Star Polymers. Macromolecules.

[34] Maruyama N., Koito T., Nishida J., Sawadaishi T., Cieren X., Ijiro K., Karthaus O., Shimomura M. (1998). Mesoscopic patterns of molecular aggregates on solid substrates. Thin Solid Film..

[35] Chen A., Blakey I., Whittaker A.K., Peng H. (2016). The influence of casting parameters on the surface morphology of PS-b -P4VP honeycomb films. J. Polym. Sci. A Polym. Chem..

[36] Hrakins W.D., Feldman A. (1921). Films. The Spreading of Liquids and the Spreading Coefficient. J. Am. Chem. Soc..

[37] Voronoi G. (1907). Nouvelles applications des paramétres continus à la théorie des forms quadratiques. J. Reine Angew. Math..

[38] Madej W., Budkowski A., Raczkowska J., Rysz J. (2008). Breath Figures in Polymer and Polymer Blend Films Spin-Coated in Dry and Humid Ambiance. Langmuir.

[39] de Leon A., Munos-Bonilla A., Fernández-Garcia M., Rodriguez-Hernández J. (2012). Breath figures method to control the topography and functionality of polymeric surfaces in porous films and microspheres. J. Polym. Sci. Part A Polym. Chem..

[40] Sun H., Li H., Wu L. (2009). Micro-patterned polystyrene surfaces surfactant-encapsulated polyoxometalate complex via breath figures. Polymer.

[41] Sakatani Y., Boisiérre C., Grosso D., Nicole L., Soler-Illia G.J.A.A., Sanchez C. (2008). Coupling Nanobuilding Block and Breath Figures Approaches for the Designed Construction of Hierarchically Templated Porous Materials and Membranes. Chem. Mater..

[42] Zhu L.-W., Ou Y., Wan L.-S., Xu Z.-K. (2014). Polystyrenes with Hydrophilic End Groups: Synthesis, Characterization, and Effects on the Self-Assembly of Breath Figure Arrays. J. Phys. Chem. B.

[43] Ji E., Pellerin V., Ehrenfeld F., Laffore A., Bousquet A., Billon L. (2017). Hierarchical honeycomb-structured films by directed self-assembly in “breath figure” templating of ionizable “clicked” PH3T-b-PMMA diblock copolymers: An ionic group/counter-ion effect on porous polymer film morphology. Chem. Commun..

