# Supplementary material for: Controlled ATRP Synthesis of Novel Linear-Dendritic Block Copolymers and Their Directed Self-Assembly in Breath Figure Arrays
Source: Polymers (Basel). 2019 Mar 21;11(3):539. doi: 10.3390/polym11030539 (PMC6473431; doi:10.3390/polym11030539)
Supplement: Supplementary file 1 [file polymers-11-00539-s001.pdf]

Supplementary Materials

Controlled ATRP Synthesis of Novel Linear-Dendritic Block Copolymers and Their Directed Self-Assembly in Breath Figure Arrays

Xin Liu [1], Tina Monzavi [1,2] and Ivan Gitsov [1,3,*]

[1] Department of Chemistry, State University of New York – College of Environmental Science and Forestry, Syracuse, NY 13210, USA; xliu70@syr.edu
[2] Department of Chemistry and Biochemistry, University of South Carolina, Columbia, SC 29208, USA; tmonzavi@email.sc.edu
[3] The Michael M. Szwarc Polymer Research Institute, Syracuse, NY 13210, USA
* Correspondence: igivanov@syr.edu; Tel.: +1-315-470-6851 (F.L.)

Received: 5 March 2019; Accepted: 19 March 2019; Published: 21 March 2019

Contents

NMR and MALDI-TOF spectra of dendritic macroinitiators…………………………….......S02

Synthesis of LDBCs by a “coupling” method…………………………….………..……..…S07

Initiator efficiency for the synthesis of linear-dendritic block copolymers by dendron initiated ATRP ….…………………………………………..………………..…………………...S09

Removal of acetonide protecting groups in the dendron block of LDBCs..………………….,S11

Additional SEM images of BF film and other miscellaneous images………….………....…….S12

References……………………………………………………………………………….,S17

NMR and MALDI-TOF spectra of dendritic macroinitiators

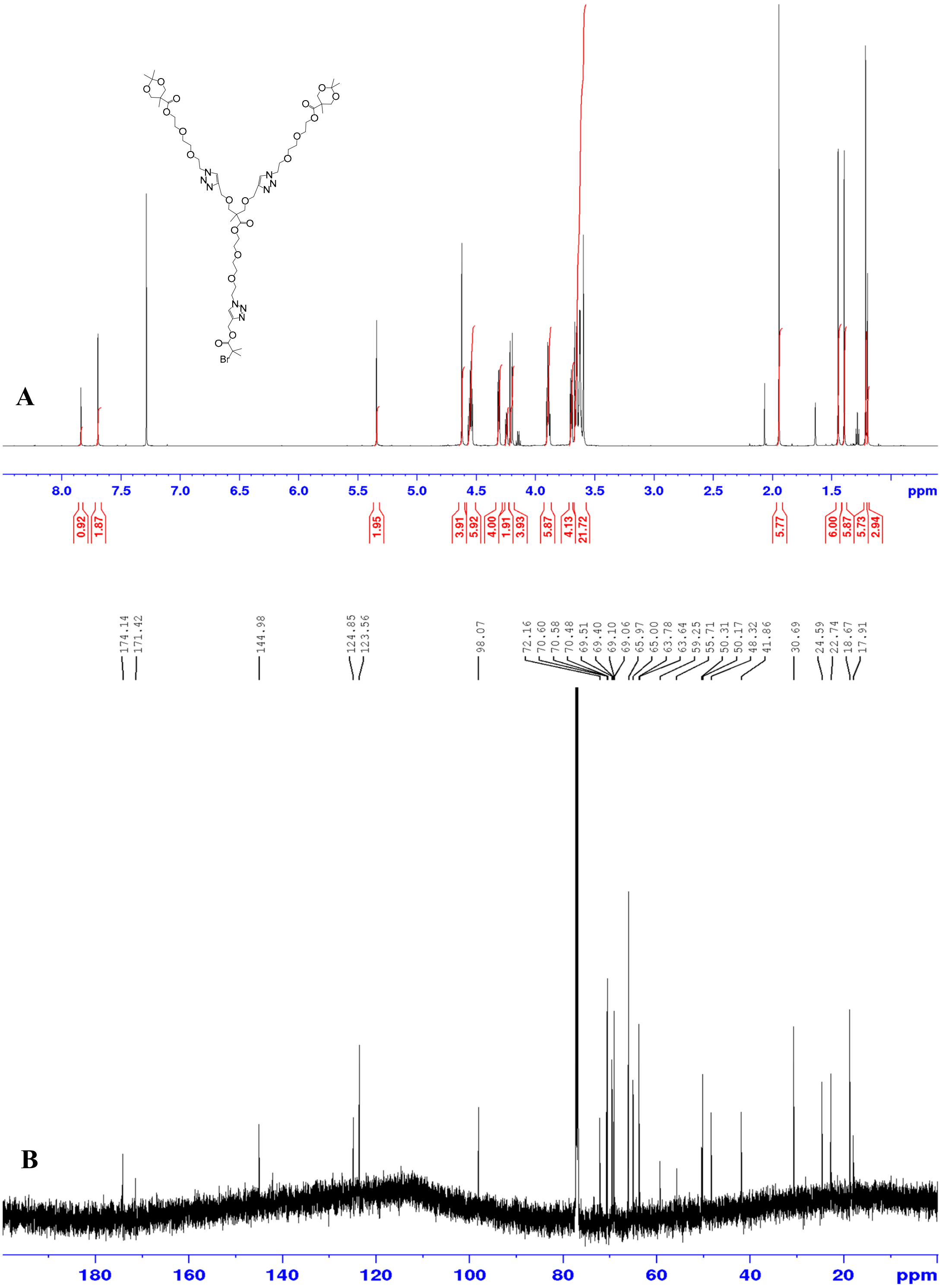

Figure S1. ^{1}H NMR (**A**) and ^{13}C NMR (**B**) of the first-generation PEE macroinitiator in $CDCl_3$

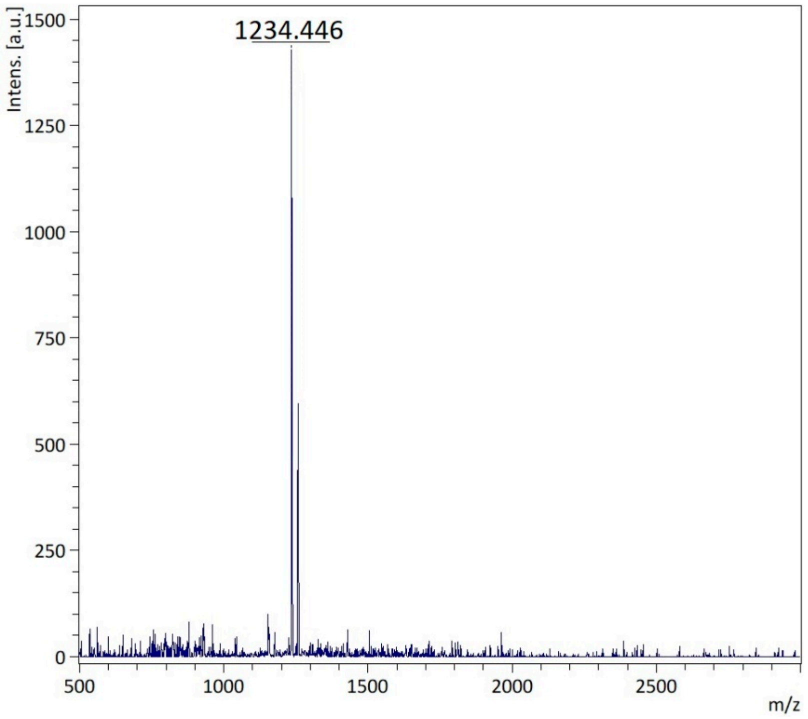

Figure S2. MALDI-TOF spectrum of first generation PEE macroinitiator

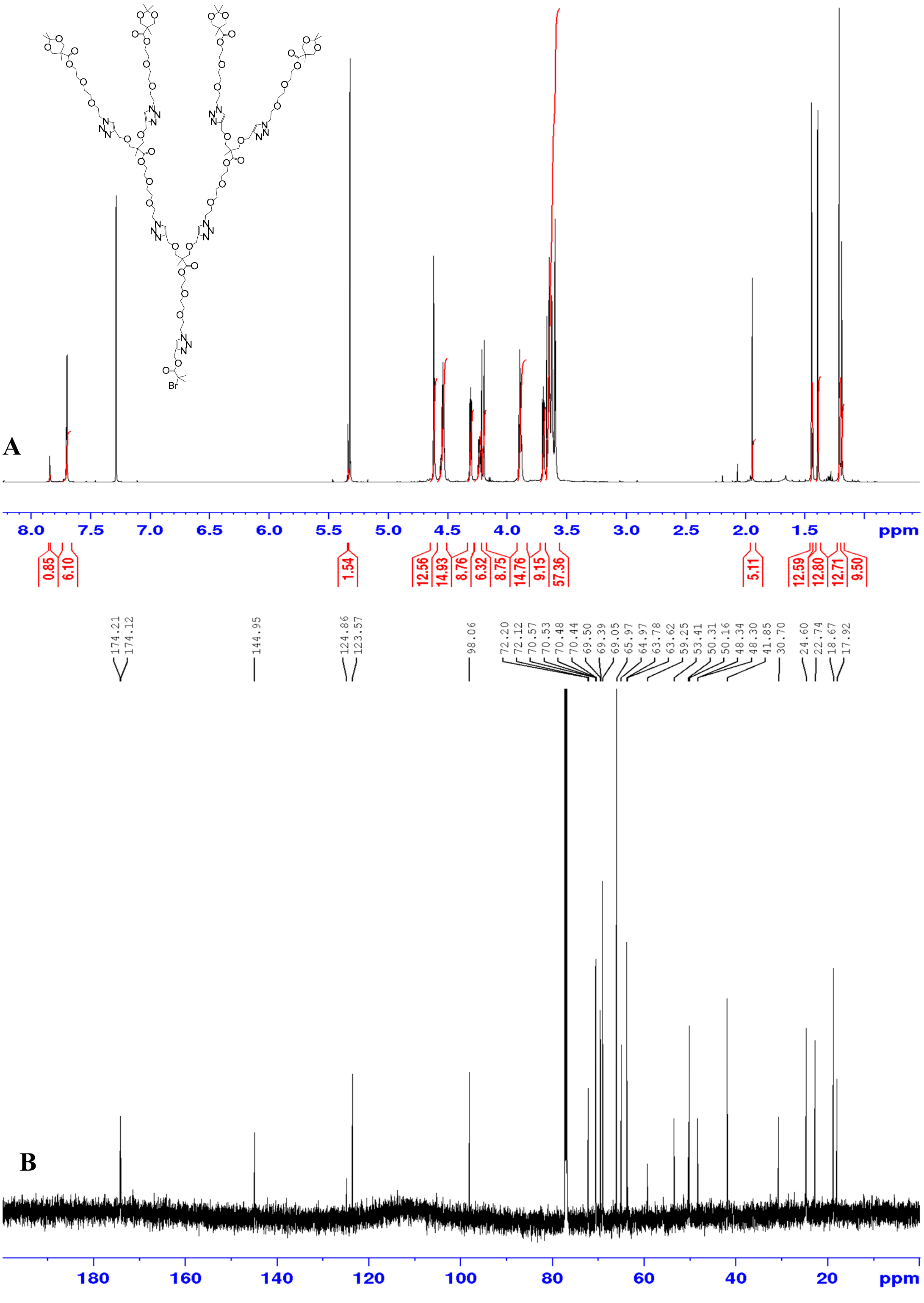

Figure S3. ^{1}H NMR (**A**) and ^{13}C NMR (**B**) of the second-generation PEE macroinitiator in $CDCl_3$

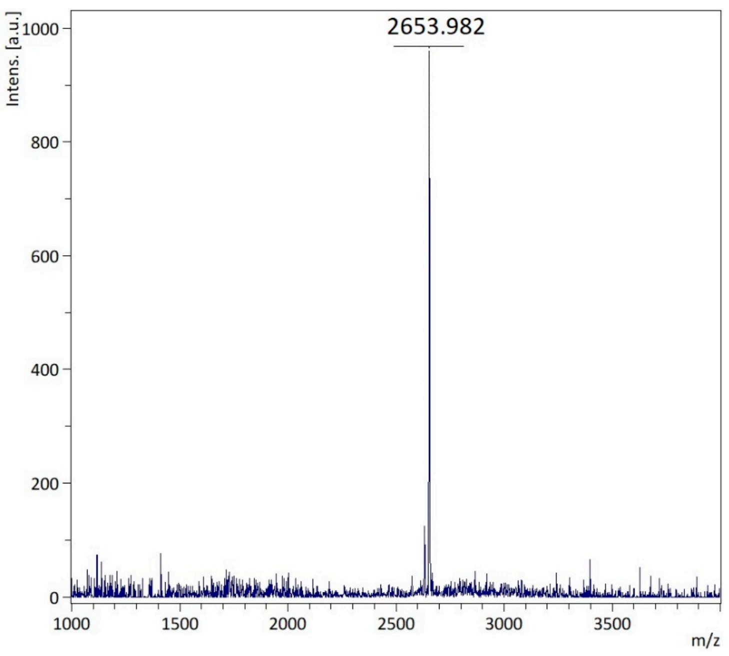

Figure S4. MALDI-TOF spectrum of second-generation PEE macroinitiator

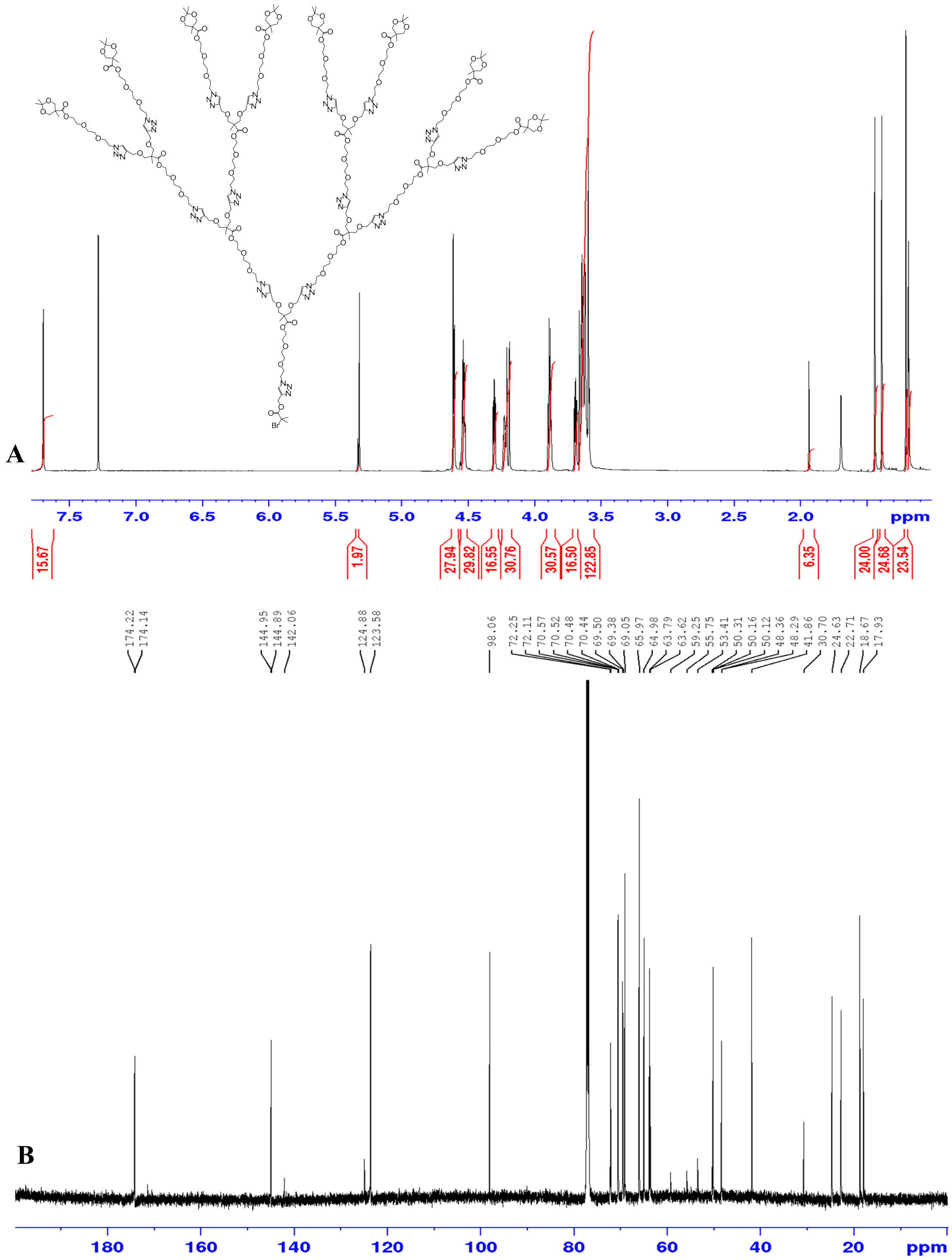

Figure S5. ^{1}H NMR (**A**) and ^{13}C NMR (**B**) of third generation PEE macroinitiator in $CDCl_3$

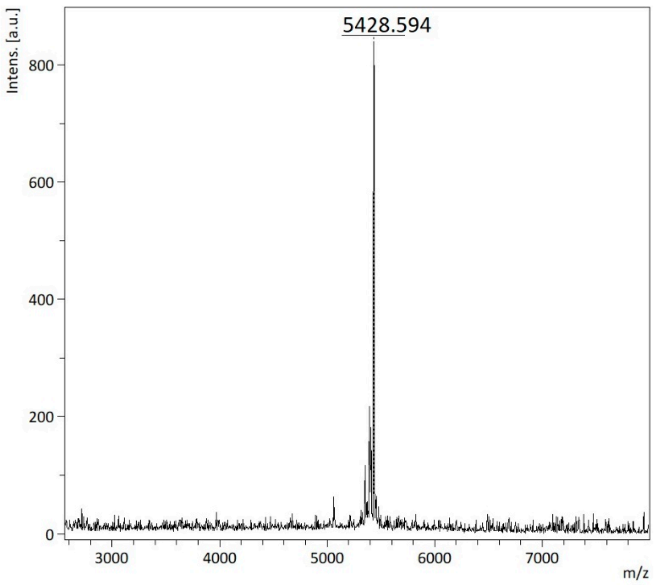

Figure S6. MALDI-TOF of third generation PEE macroinitiator

Synthesis of LDBC by a "coupling" method. Poly(styrene) with alkyne end group, namely PSt-A 3.2k, was synthesized by ATRP using propargyl 2-bromoisobutyrate as initiator [1]. PSt-A (50 mg, 0.015 mmol), LG2-N_3(50 mg, 0.021 mmol) and PMDTA (10 µL) were dissolved in 2 mL DMF. The solution was degassed by 3 freeze - thaw cycles. After warming to room temperature, the solution was transferred into a flask charged with 10 mg CuBr. The solution was stirred at room temperature overnight. The resulting solution was diluted with 10 mL of water and extracted with DCM. The DCM product was precipitated into methanol and analyzed by SEC, Figure S7. The "bump" appearing in the high molecular mass region indicates the formation of "oxidative" coupling product (Scheme S1) similar to what was previously observed in Janus dendrimer synthesis by "coupling" method [2].

Scheme S1. Synthesis of LDBC by a "coupling" method

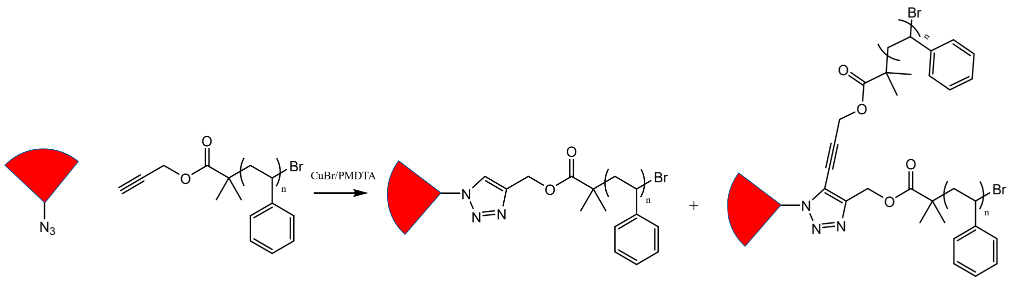

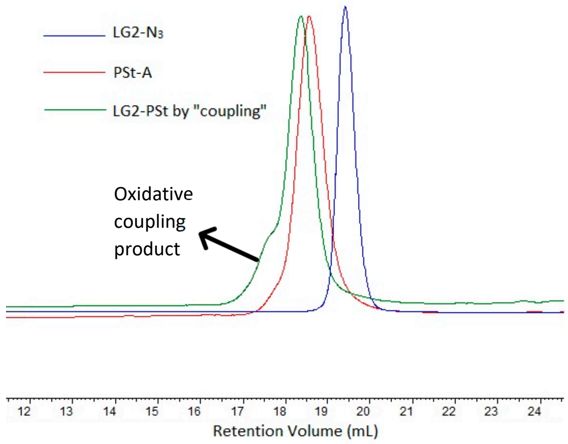

Figure S7. SEC traces of LDBC prepared by "coupling" method. The obtained LDBC is a mixture of normal "click" and oxidative coupling products.

Initiator efficiency for the synthesis of linear-dendritic block copolymers by dendron initiated ATRP.

The dendritic marcoinitiator efficiency f_{LGn} is calculated as a ratio of the mass of the dendron incorporated into the LDBC to the mass of the dendron initiator used. For example, 24 mg of LG2-i was used to produce 80.5 mg LG2-PSt 10k. The mass of the dendron incorporated into the LDBC is 80.5mg*F_{PEE}(23%) = 18.5 mg. Thus, the f_{LG2} in this experiment is 18.5/24 = 77 %. F_{PEE} is the mass fraction of the PEE dendron in LDBC determined by ^{1}H NMR using the integral intensity of methyl group on the peripheral 2,2-bis(hydroxymethyl) propionic acid unit (marked as "m1, m2, m3" in Figure S8) and aromatic protons on poly(styrene).

f_{LG2} could be also calculated by ratio of the theoretical molecular mass of polystyrene chain to the observed molecular mass, in which the theoretical molecular mass could be calculated by:

$$M_{theor} = \frac{[St]_0}{[LG2-i]_0} \times conversion \times 104$$

$$f_{LG2} = M_{theor}/M_{obs} \times 100,$$

where $[M]_0$ and $[I]_0$ are the original mole of styrene and initiator, respectively, 104 is the molecular mass of styrene monomer. In the above experiment, conversion is 14.5 %. The theoretical M_w of poly(styrene) is 6032 g/mol. Comparing to observed M_w 8320 (^{1}H NMR, degree of polymerization is 80), f_{LG2} is calculated to be 72.5 %, which is slightly lower than the method mentioned above.

The acetonide protecting groups are removed by treating the LDBCs with TFA. Briefly, 10 mg of LDBC was dissolved in 2 mL DCM and 1 mL of TFA was added to the above solution. The reaction mixture was stirred at room temperature for 30 min before the solvents were evaporated. The obtained LDBCs were dissolved into $CHCl_3$ and residual TFA was neutralized by $NaHCO_3$. The resulted solution was washed with water and dried. The deprotected LDBCs, namely deLGn-PSt *Mn*k, was obtained by evaporating the solvent.

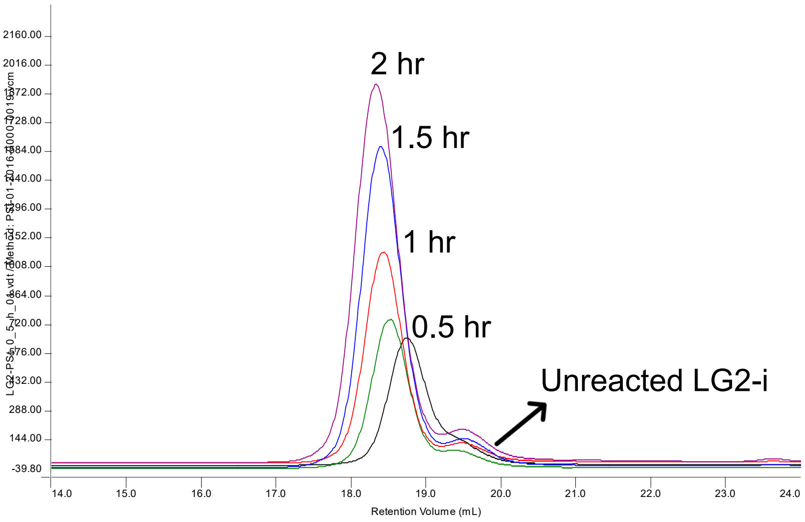

Figure S8. SEC monitoring the LG2-i initiated polymerization progress with time. The small peak appearing at lower molecular mass region is the unreacted dendron initiator.

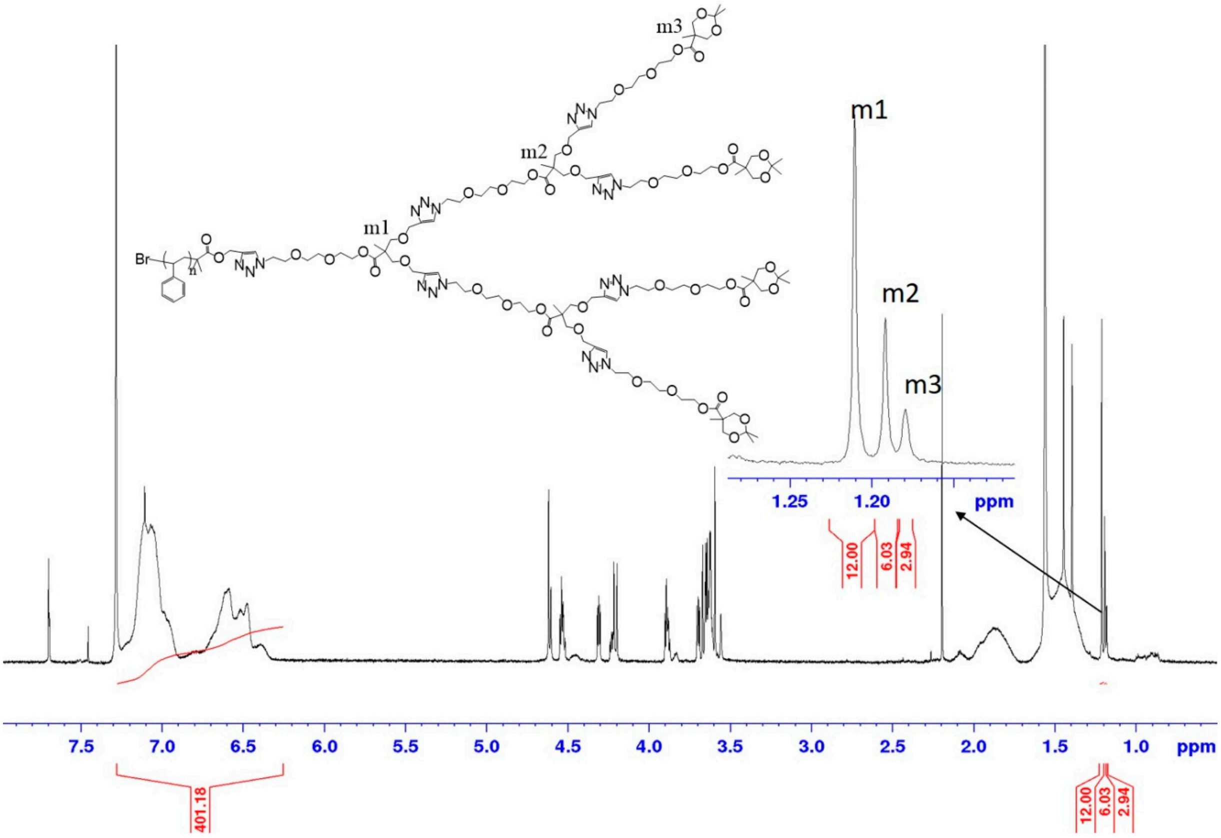

Figure S9. ^{1}H NMR spectrum of LG2-PSt 10k as an example how to calculate F_{PEE}.

Removal of acetonide protecting groups in the dendron block of LDBCs

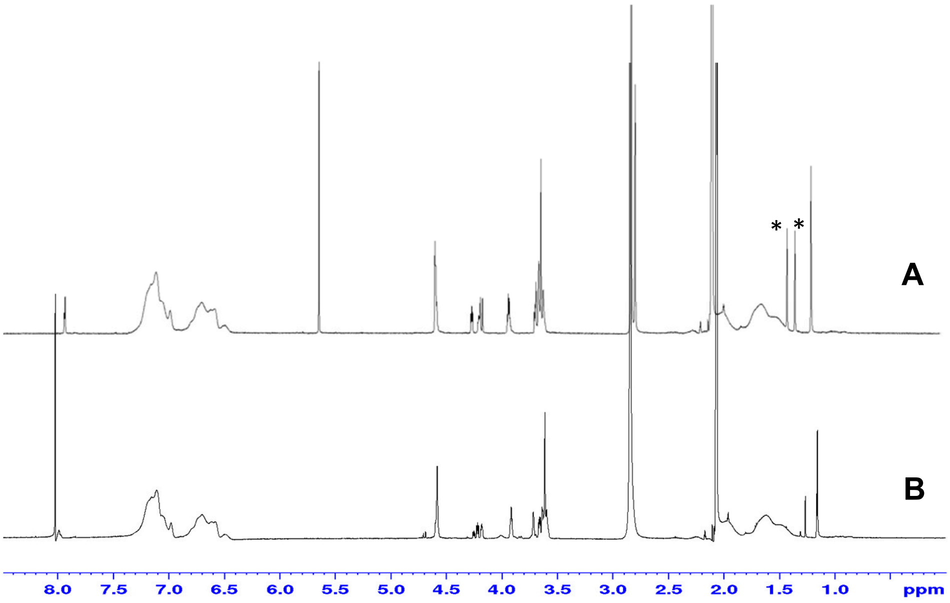

Figure S10. NMR of LG3-PSt 20k before (**A**) and after (**B**) the acetonide protecting group is removed as highlighted. The signals of CH_3 groups on acetonide at 1.44 and 1.38 (marked as *) ppm are completely removed after deprotection step.

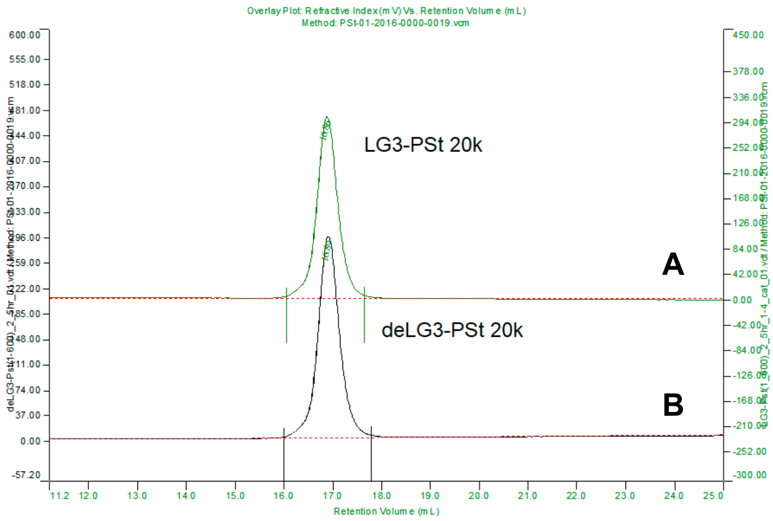

Figure S11. SEC traces of LG3-PSt 20k before (**A**) and after deprotection (**B**) (deLG3-PSt 20k).

Additional SEM images and miscellaneous

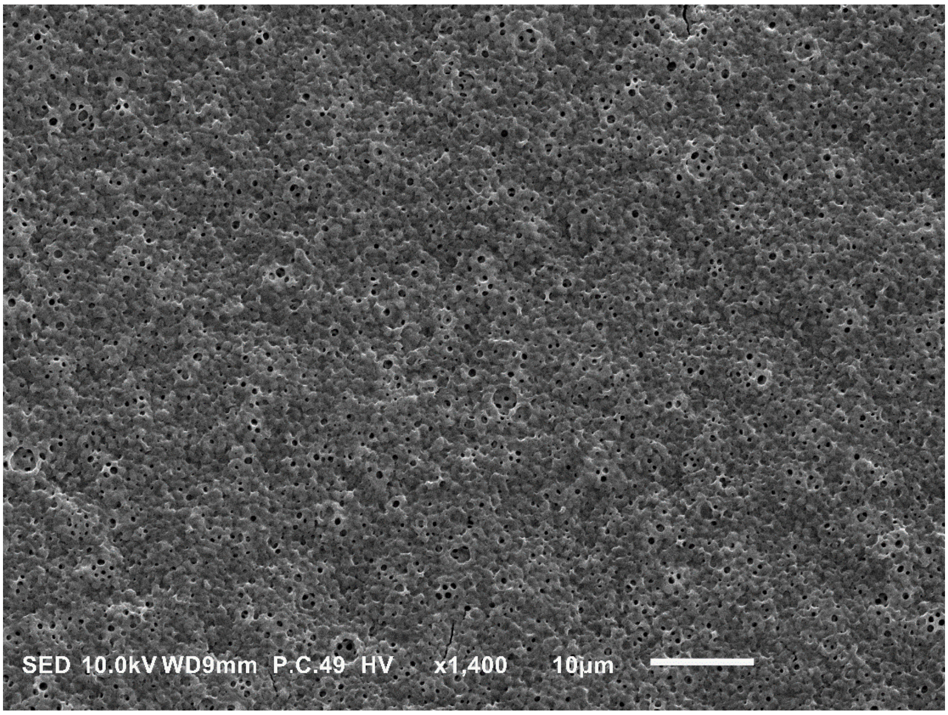

Figure S12. SEM image of BF film prepared by LG1-PSt 4.9k. Rough surface with 0.2-0.6 μm pores irregularly distributed.

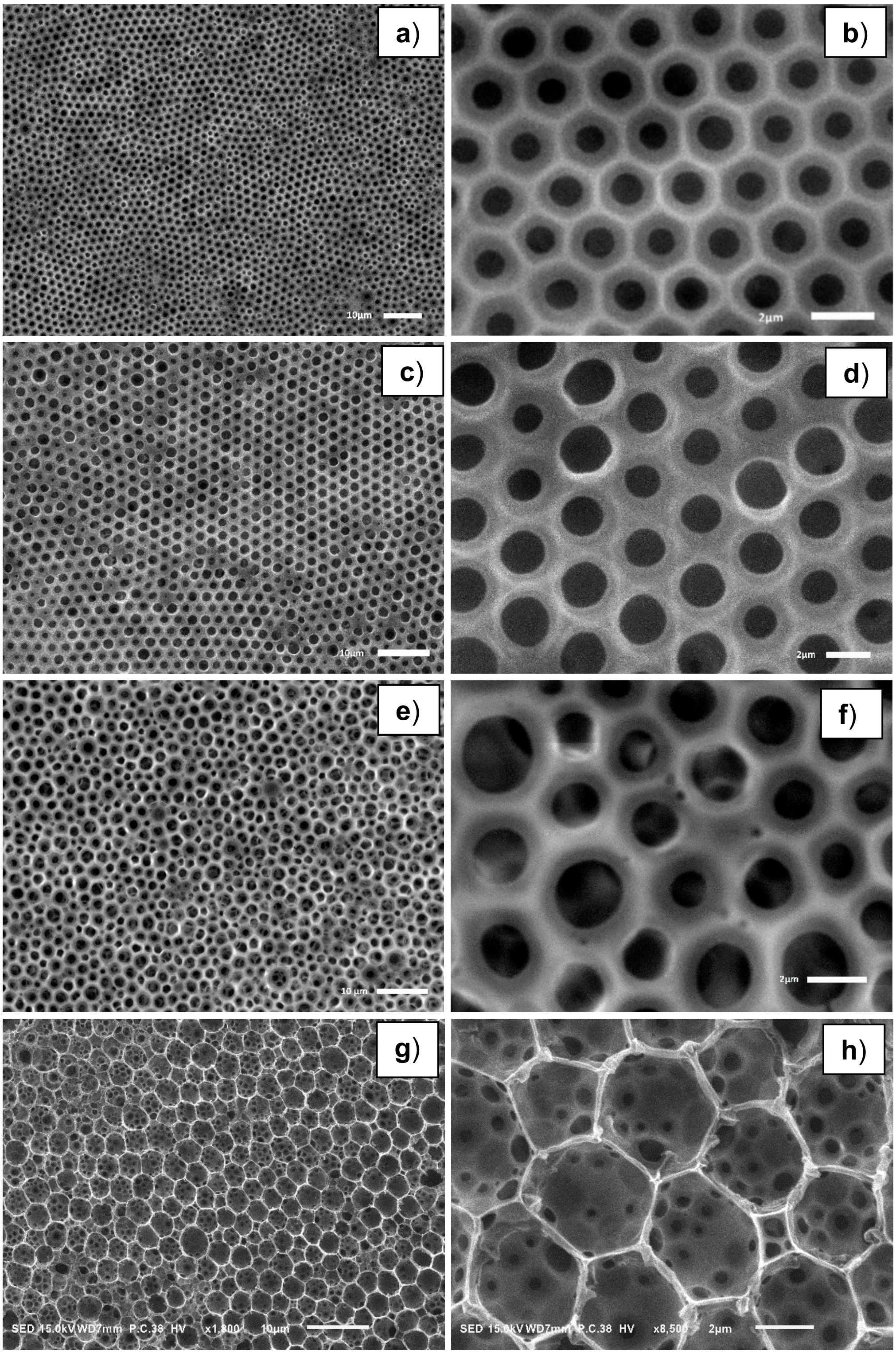

Figure S13. SEM images of BF films prepared by the second generation LDBCs with acetonide end groups: (**a**, **b**) LG2-PSt 23k; (**c**, **d**) LG2-PSt 14k; (**e**, **f**) LG2-PSt 10k, major portion of the film, (**g**, **h**) LG2-PSt 10k, edge of the film.

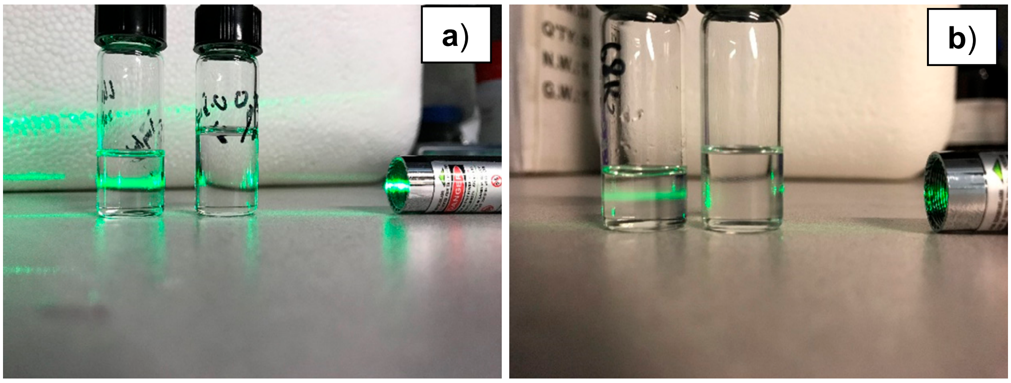

Figure S14. Tyndall effect of inverse micelles formed by LDBCs with hydroxyl end groups in chloroform (vial on the left side) and inverse micelles are dissolved by adding 25 % THF (vial on the right side): (**a**) deLG2-PSt 23k; (**b**) deLG3-PSt 68k.

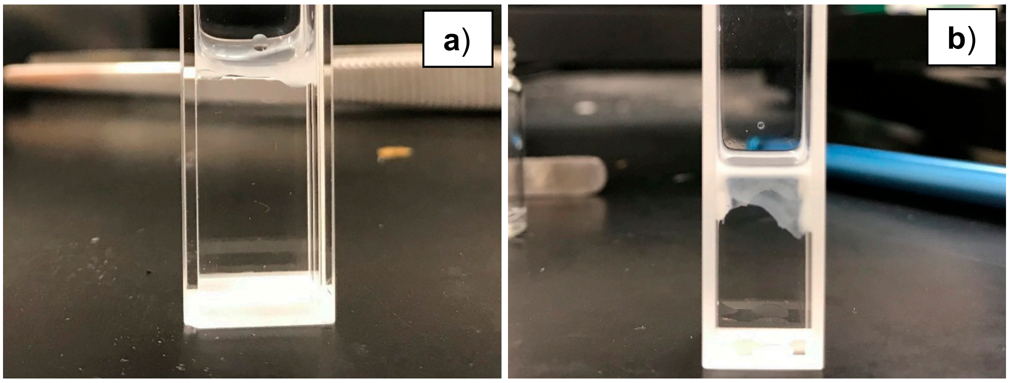

Figure S15. Emulsion formed by placing LDBCs with hydroxyl end groups in THF/chloroform solution added to water:(**a**) deLG2-PSt 23k; (**b**) deLG3-PSt 68k

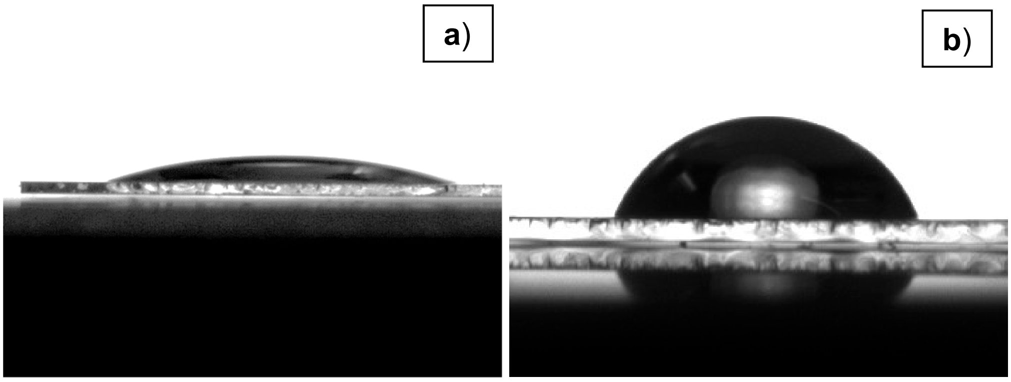

Figure S16. Contact angle measurement of water droplet on the polymer film prepared by air dry film drop casted on glass surface in different solvents: (**a**) pure chloroform; (**b**) 1:3 THF/chloroform. The LDBC used is deLG3-PSt 68k.

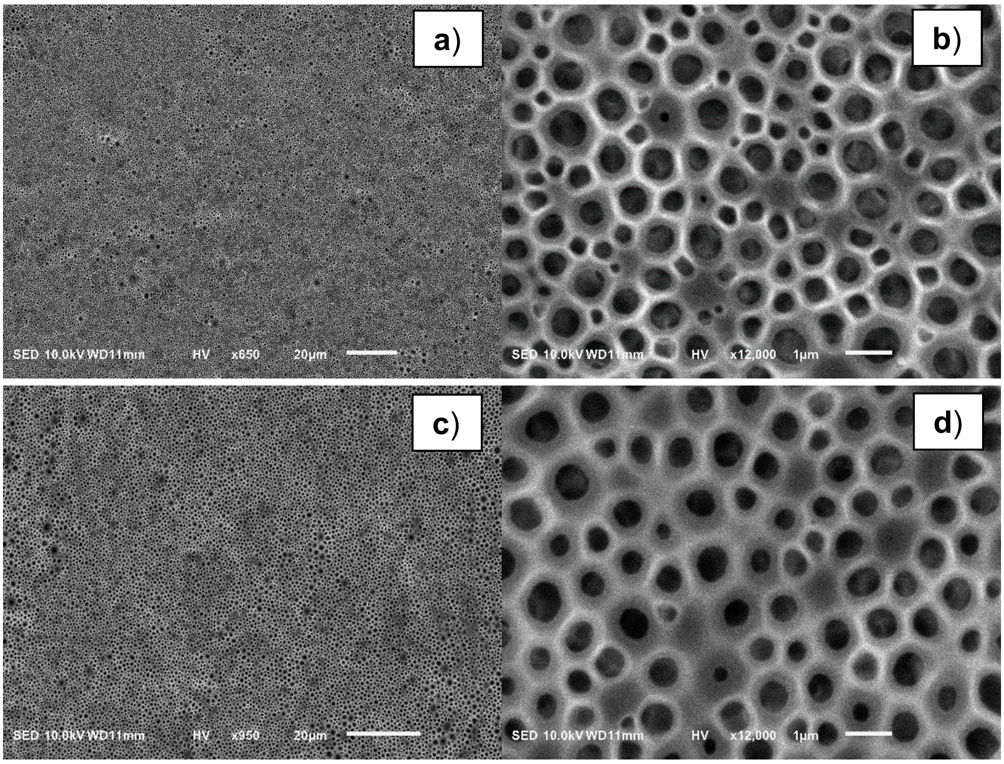

Figure S17. SEM images of BF films prepared by deLG3-PSt 68k in mix solvents: (**a**,**b**) 1:3 acetone/chloroform; (**c**,**d**) 1:3 acetonitrile/chloroform

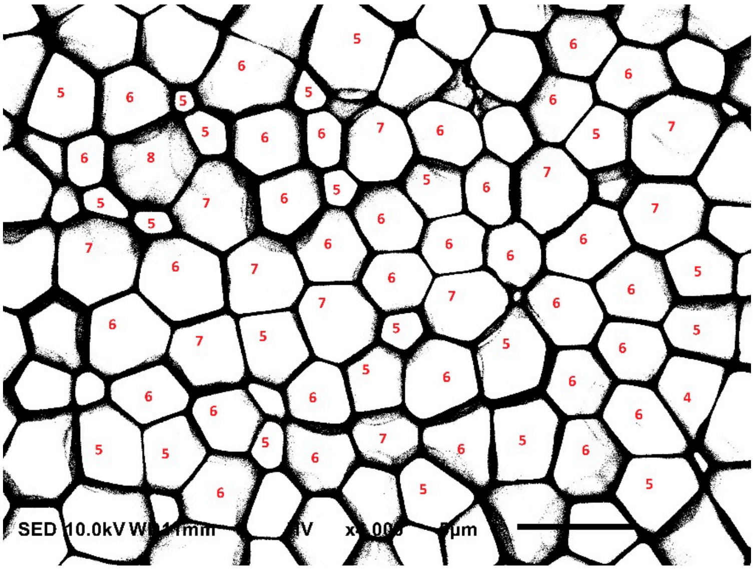

Figure S18. Binary image obtained from Figure 8 using ImageJ to estimate Voronoi number.

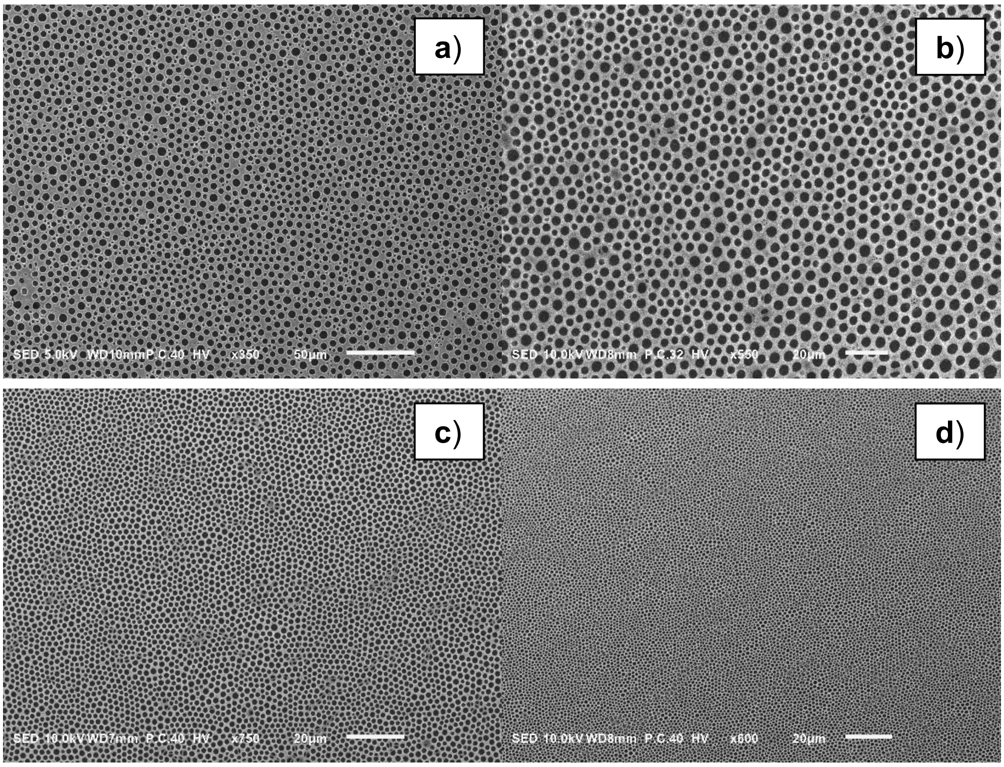

Figure S19. SEM images of BF films prepared by Homo PSt mixed with deLG2-PSt 23k at different concentrations: a) 0.1 wt %; b) 0.2 wt %; c) 0.4 wt %; d) 0.6 wt %.

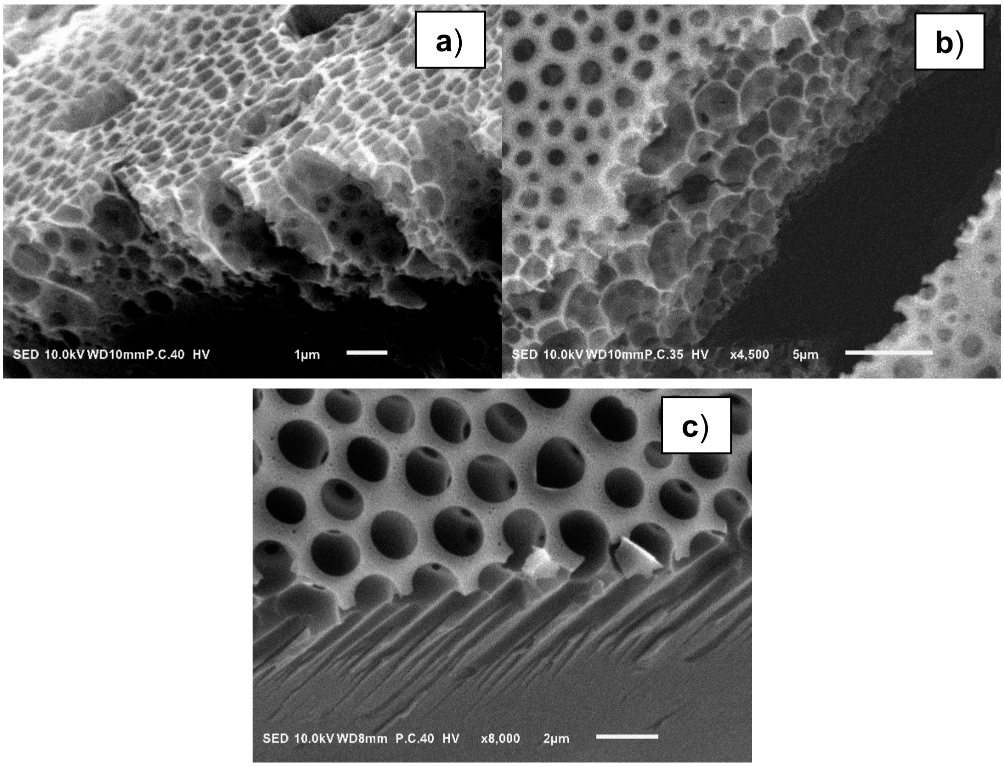

Figure S20. SEM images of fractured BF films produced by: (**a**) LG2-PSt 23k; (**b**) deLG2-PSt 23k; (**c**) deLG2-PSt 23k and homo poly(styrene) blend

References

1. B.A. Laurent, S.M. Grayson, An Efficient Route to Well-Defined Macrocyclic Polymers via "Click" Cyclization. J. Am. Chem. Soc. 2006**,** 128(13), 4238-4239.

2. X. Liu, I. Gitsov, Thermosensitive Amphiphilic Janus Dendrimers with Embedded Metal Binding Sites. Synthesis and Self-Assembly. Macromolecules 2018**,** 51(14), 5085-5100.
